# Supplementary material for: Indole- and Pyrazole-Glycyrrhetinic Acid Derivatives as PTP1B Inhibitors: Synthesis, In Vitro and In Silico Studies
Source: Molecules. 2021 Jul 20;26(14):4375. doi: 10.3390/molecules26144375 (PMC8308021; doi:10.3390/molecules26144375)
Supplement: Supplementary file 1 [file molecules-26-04375-s001.zip › molecules-1290170-supplementary.pdf]

Supplementary Data

Indole- and Pyrazole-Glycyrrhetinic Acid Derivatives as PTP1B Inhibitors: Synthesis, In Vitro and In Silico Studies

Ledy De-la-Cruz-Martínez [1,2,†], Constanza Duran-Becerra [1,†], Martin González-Andrade [3], José C. Páez-Franco [4], Juan Manuel Germán-Acacio [4], Julio Espinosa-Chávez [5], J. Martin Torres-Valencia [6], Jaime Pérez-Villanueva [1], Juan Francisco Palacios-Espinosa [1], Olivia Soria-Arteche [1] and Francisco Cortés-Benítez [1,*]

[1] Departamento de Sistemas Biológicos, División de Ciencias Biológicas y de la Salud, Universidad Autónoma Metropolitana–Xochimilco (UAM–X), 04960 Ciudad de México, Mexico; 2193800101@alumnos.xoc.uam.mx (L.D.-l.-C.-M.); 2163025430@alumnos.xoc.uam.mx (C.D.-B.); jpvillanueva@correo.xoc.uam.mx (J.P.-V.); jpalacios@correo.xoc.uam.mx (J.F.P.-E.); soriao@correo.xoc.uam.mx (O.S.-A.)

[2] Maestría en Ciencias Farmacéuticas, División de Ciencias Biológicas y de la Salud, Universidad Autónoma Metropolitana–Xochimilco (UAM–X), 04960 Ciudad de México, Mexico

[3] Departamento de Bioquímica, Facultad de Medicina, Universidad Nacional Autónoma de México, 04510 Ciudad de México, Mexico; martin@bq.unam.mx

[4] Red de Apoyo a la Investigación, Universidad Nacional Autónoma de México e Instituto Nacional de Ciencias Médicas y Nutrición Salvador Zubirán, 14000 Ciudad de México, Mexico; paez@cic.unam.mx (J.C.P.-F.); jmga@cic.unam.mx (J.M.G.-A.)

[5] Instituto de Investigaciones Químico Biológicas, Universidad Michoacana de San Nicolás de Hidalgo, 58030 Michoacán, Mexico; julioespinosa30@hotmail.com

[6] Área Académica de Química, Universidad Autónoma del Estado de Hidalgo, 42184 Hidalgo, Mexico; jmartin@uaeh.edu.mx

† These authors contributed equally to this work.

* Correspondence: jcortesb@correo.xoc.uam.mx; Tel.: +52-55-5483-4000 (ext. 7259)

Table with ^{1}H and ^{13}C NMR chemical shifts (δ) of compounds **4f** and **5f**. ^{1}H NMR and ^{13}C NMR spectra of final compounds. Spectra resulting from different NMR experiments (NOESY, COSY, HSQC and HMBC) of compounds **4f** and **5f**. UPLC–ESI–MS data of final compounds are available.

Table S1. ^{1}H and ^{13}C NMR chemical shifts (δ) of compounds **4f** and **5f**

H/C number[a]	^{1}H NMR δ(ppm)[a]	^{13}C NMR δ(ppm)[a]	H/C number[b]	^{1}H NMR δ(ppm)[b]	^{13}C NMR δ(ppm)[b]
1	2.3 and 4.0	37.5	1	2.17 and 3.55	37.03
2	-	108.4	2	-	114.40
3	-	142.07	3	-	145.79
4	-	34.2	4	-	34.45
5	1.44	53.1	5	1.32	53.58
6	1.39 and 1.72	18.68	6	1.52 and 1.68	18.05
7	1.55 and 1.80	32.2	7	1.42 and 1.70	31.80
8	-	45.5	8	-	44.71
9	-	60.5	9	2.57	59.76
10	-	38.1	10	-	37.75
11	-	199.8	11	-	198.85
12	5.80	129.01	12	5.47	127.46
13	-	169.30	13	-	170.36
14	-	44.01	14	-	44.84
15	1.28 and 2.08	27.74	15	1.35 and 1.80	26.40
16	1.07 and 2.06	26.63	16	0.99 and 2.10	26.04
17	-	31.10	17	-	31.46
18	-	48.3	18	2.06	48.25
19	1.69 and 2.02	41.10	19	1.69 and 2.11	41.02
20	-	43.5	20	-	43.31
21	1.55 and 1.79	32.06	21	1.33 and 1.83	30.59
22	1.37 and 1.44	37.92	22	1.25 and 1.36	37.70
23	1.27	23.51	23	0.97	29.54
24	1.33	31.14	24	0.94	22.74
25	1.18	16.16	25	1.04	15.63
26	1.24	28.73	26	1.08	18.12
27	1.23	18.50	27	1.08	28.03
28	0.87	28.57	28	0.75	28.18
29	1.42	23.59	29	1.35	23.07
30	-	182.06	30	-	177.76
3a′	-	137.75	1′	-	145.95
4′	7.79	116.39	2′	7.86	126.12
5′	-	121.58	3′	7.61	130.19
6′	7.34	118.00	4′	7.86	126.15
7′	7.34	110.38	5′	7.61	129.74
7a′	-	127.86	6′	-	126.08
CF_3	-	126.10	CF_3	-	122.75
NH	7.91	-	Pyrazole-CH	7.36	138.69

[a] Values determined for compound **4f**
[b] Values determined for compound **5f**

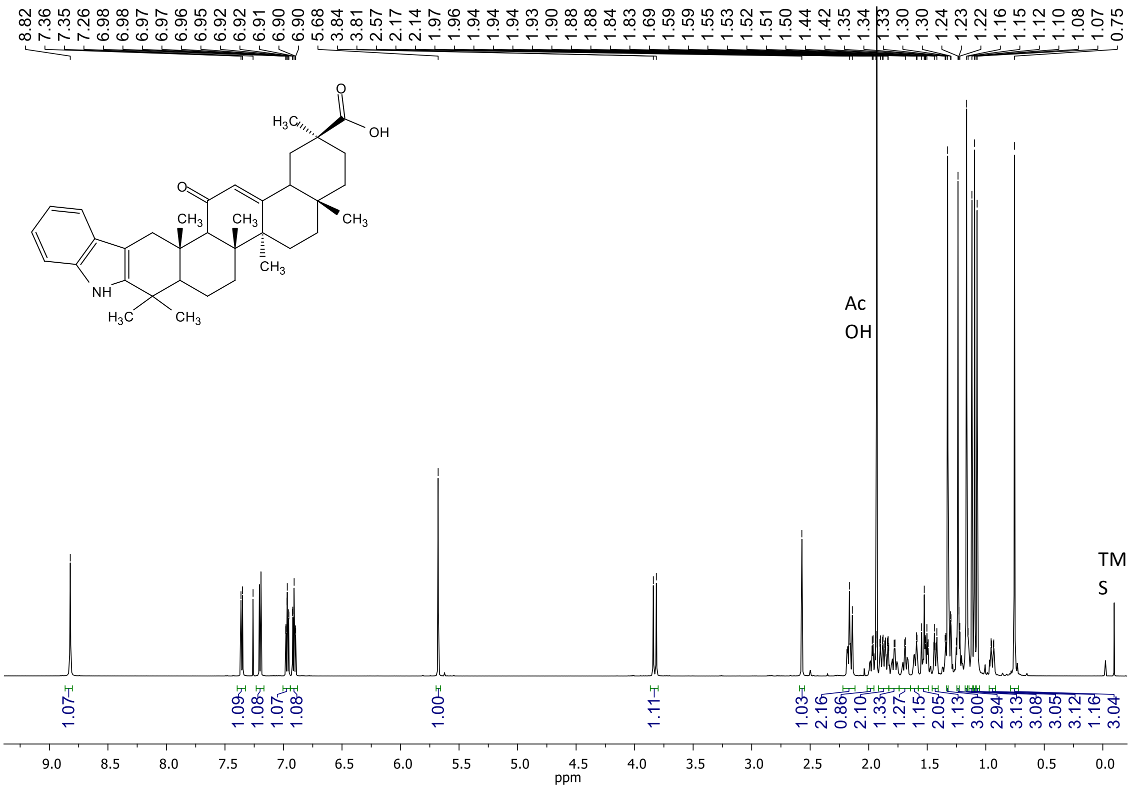

Figure S1. ^{1}H NMR (600 MHz) spectrum of compound **4a** in $CDCl_3$

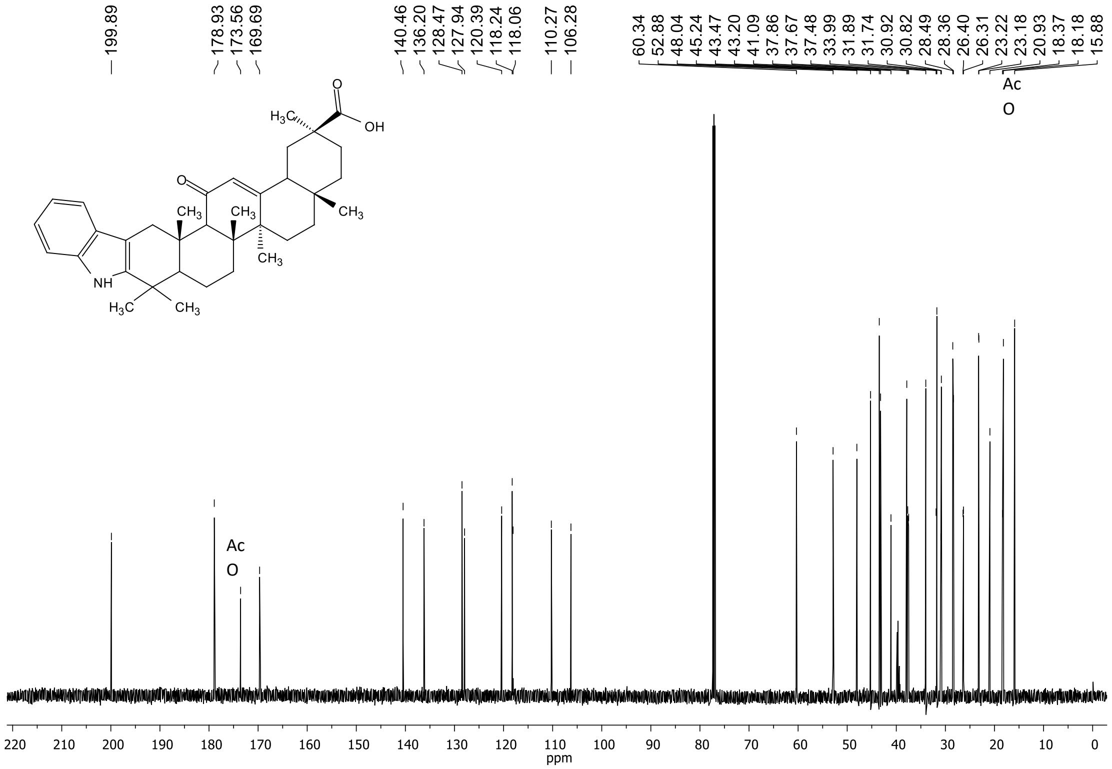

Figure S2. ^{13}C NMR (151 MHz) spectrum of compound **4a** in $CDCl_3$

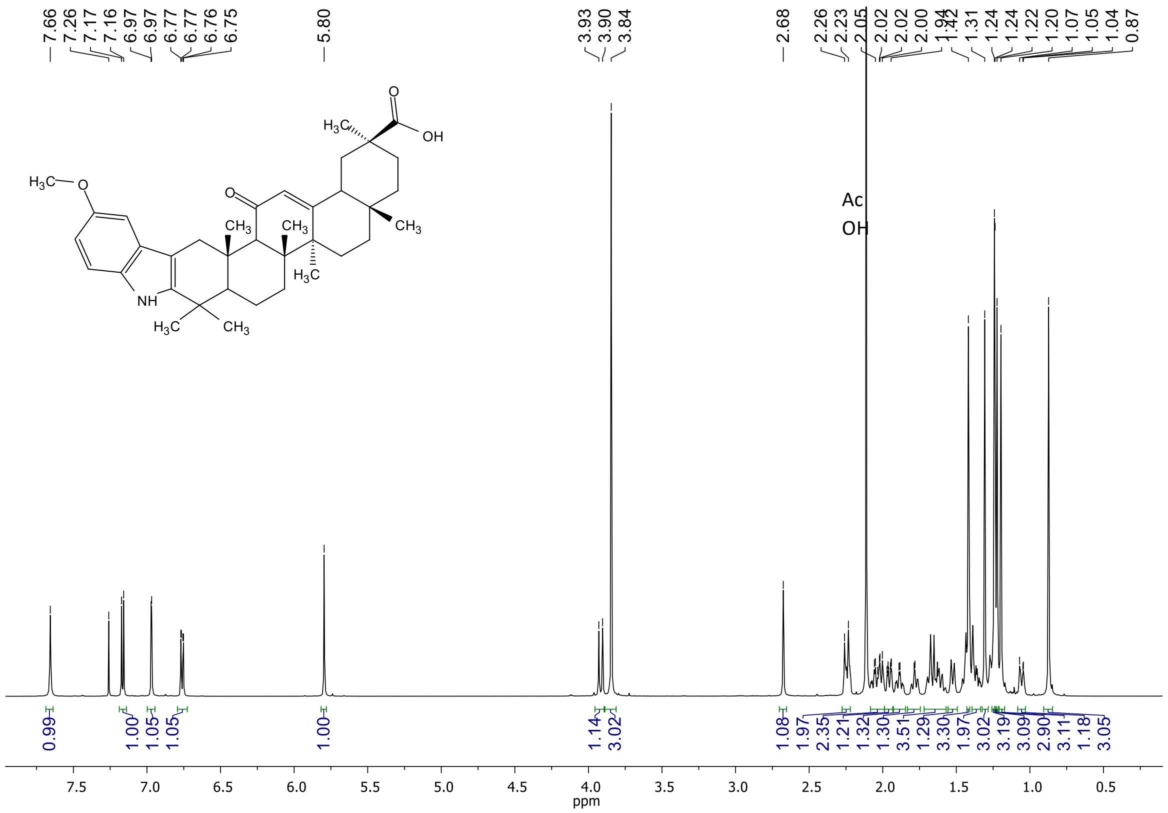

Figure S3. ^{1}H NMR (600 MHz) spectrum of compound **4b** in $CDCl_3$

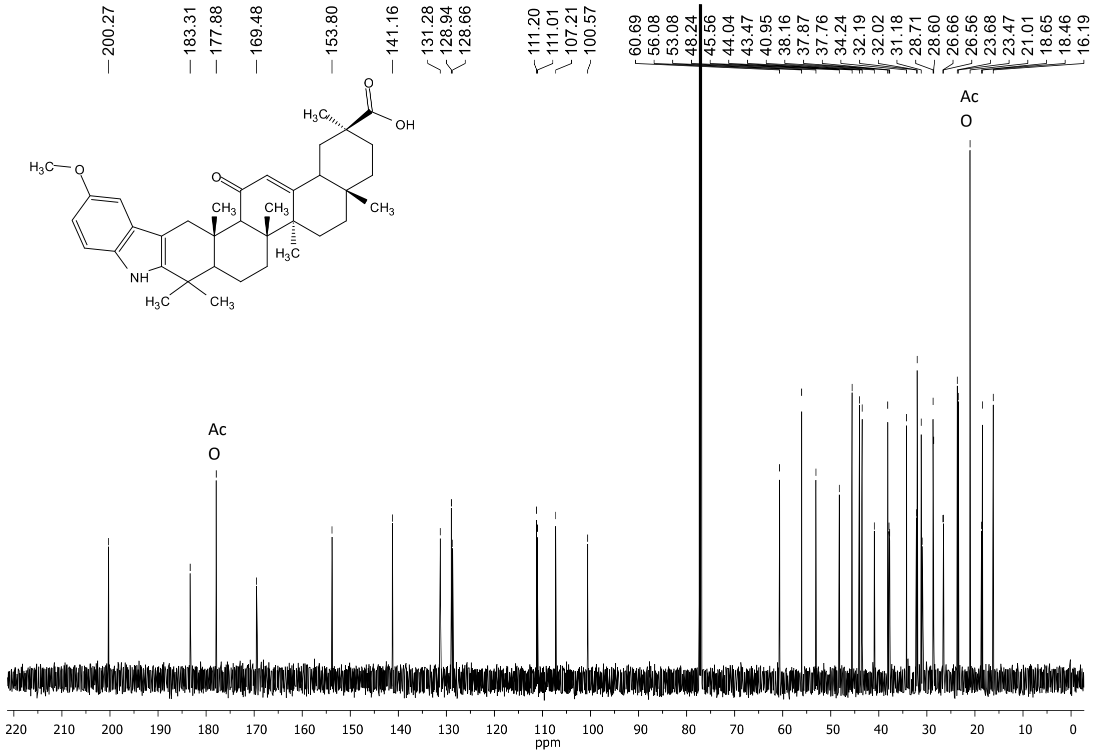

Figure S4. ^{13}C NMR (151 MHz) spectrum of compound **4b** in $CDCl_3$

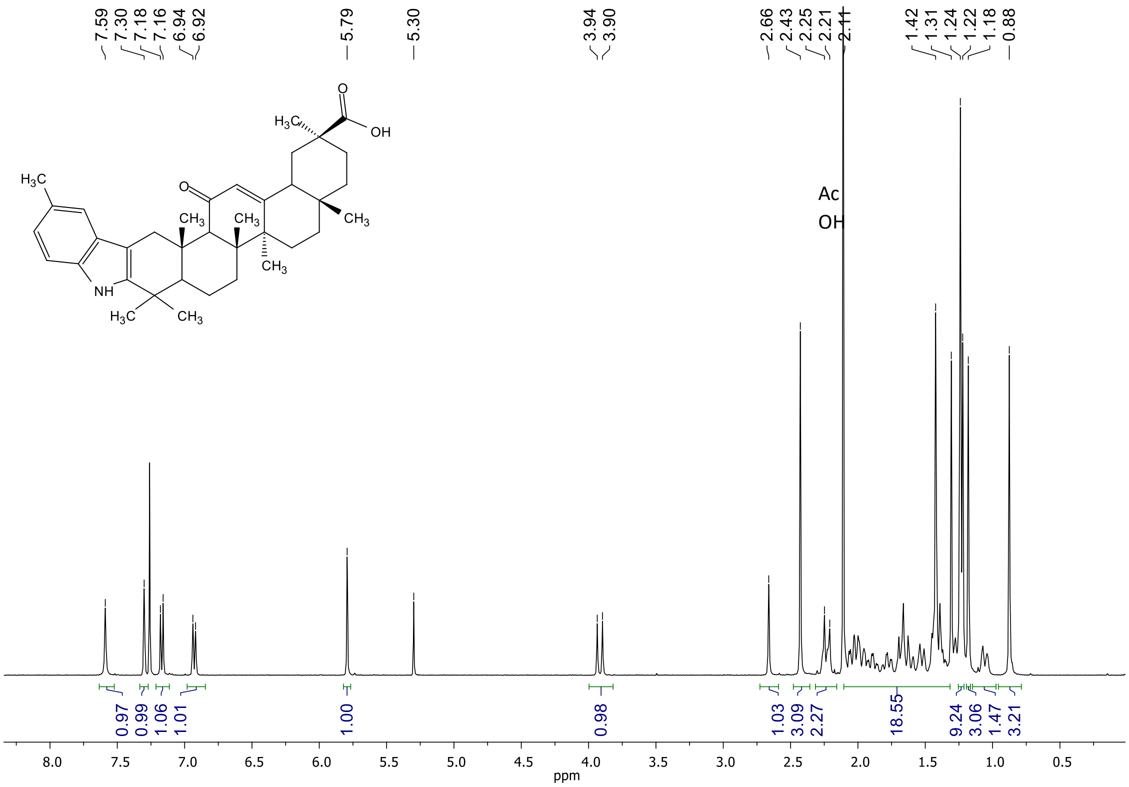

Figure S5. ^{1}H NMR (600 MHz) spectrum of compound **4c** in $CDCl_3$

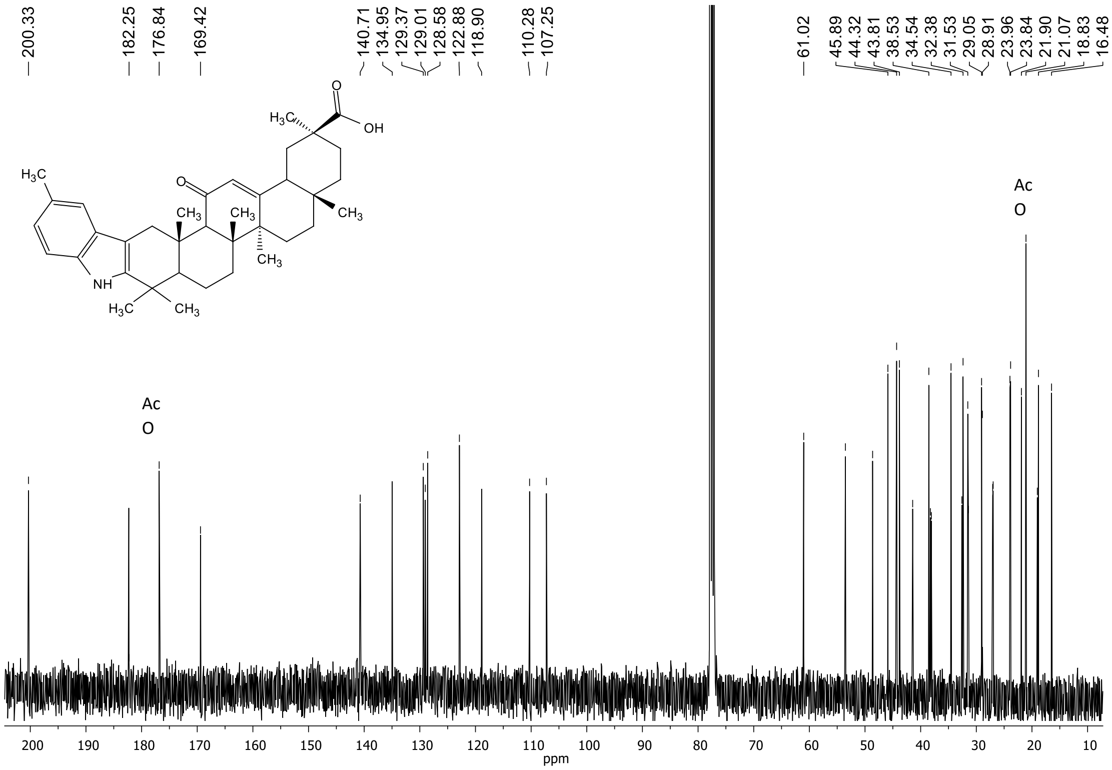

Figure S6. ^{13}C NMR (151 MHz) spectrum of compound **4c** in $CDCl_3$

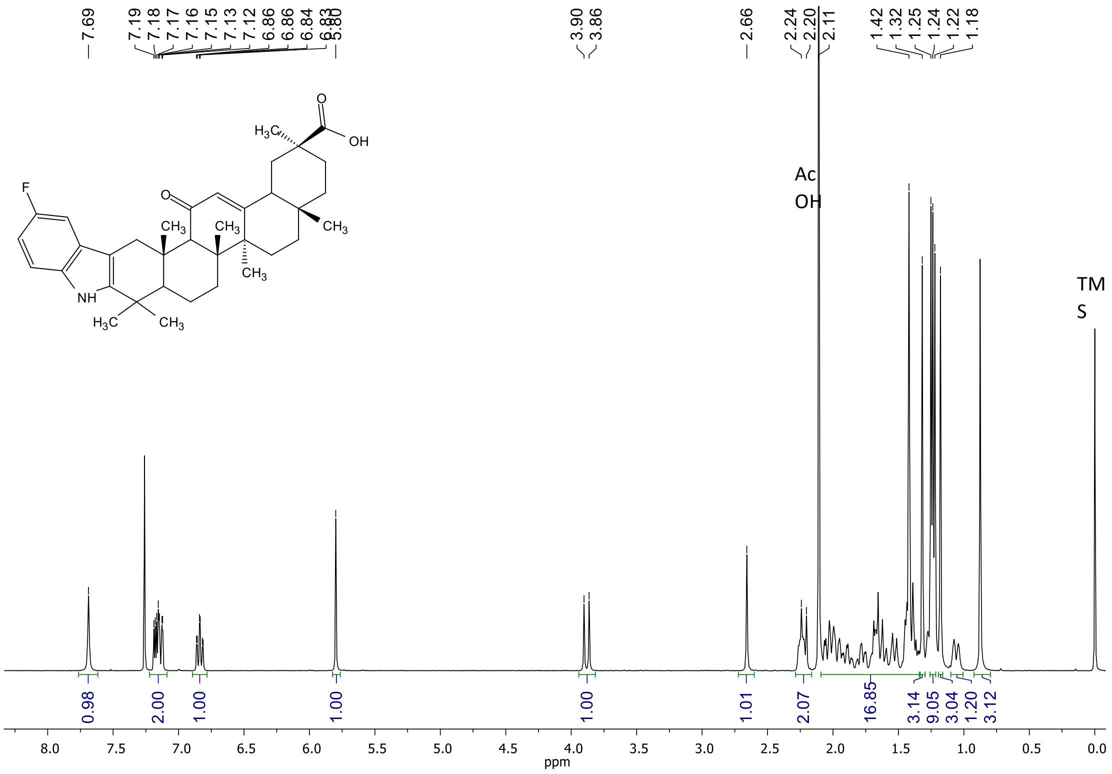

Figure S7. ^{1}H NMR (600 MHz) spectrum of compound **4d** in $CDCl_3$

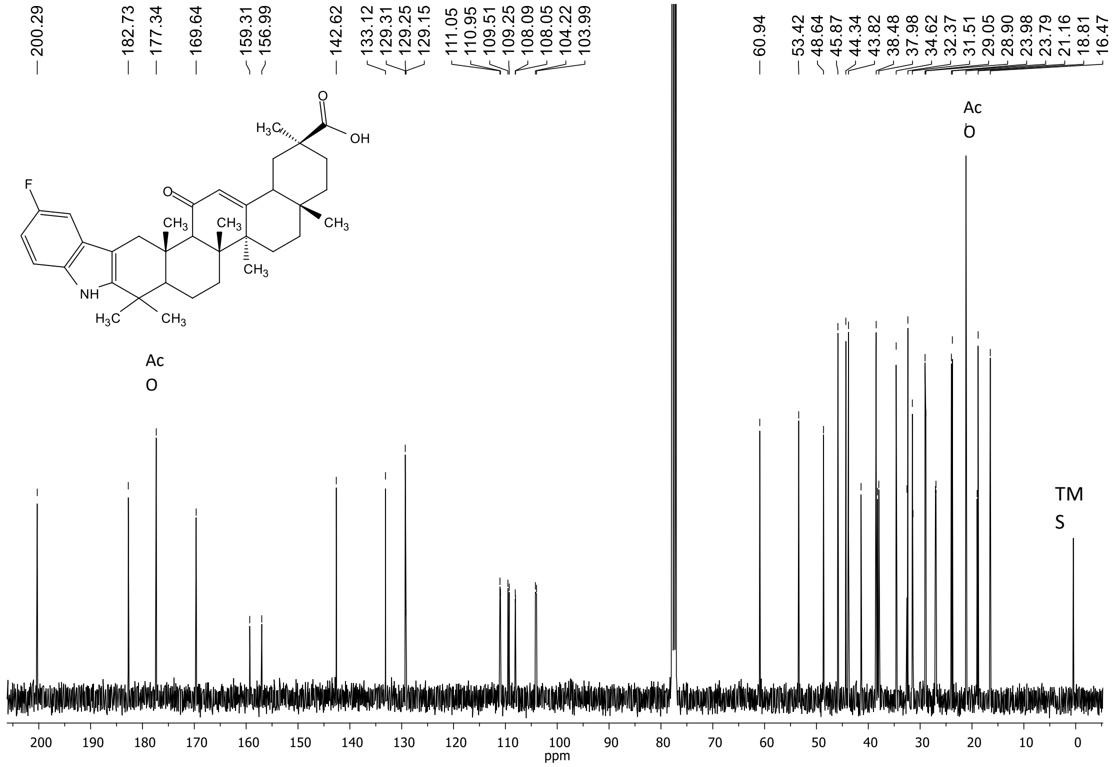

Figure S8. ^{13}C NMR (151 MHz) spectrum of compound **4d** in $CDCl_3$

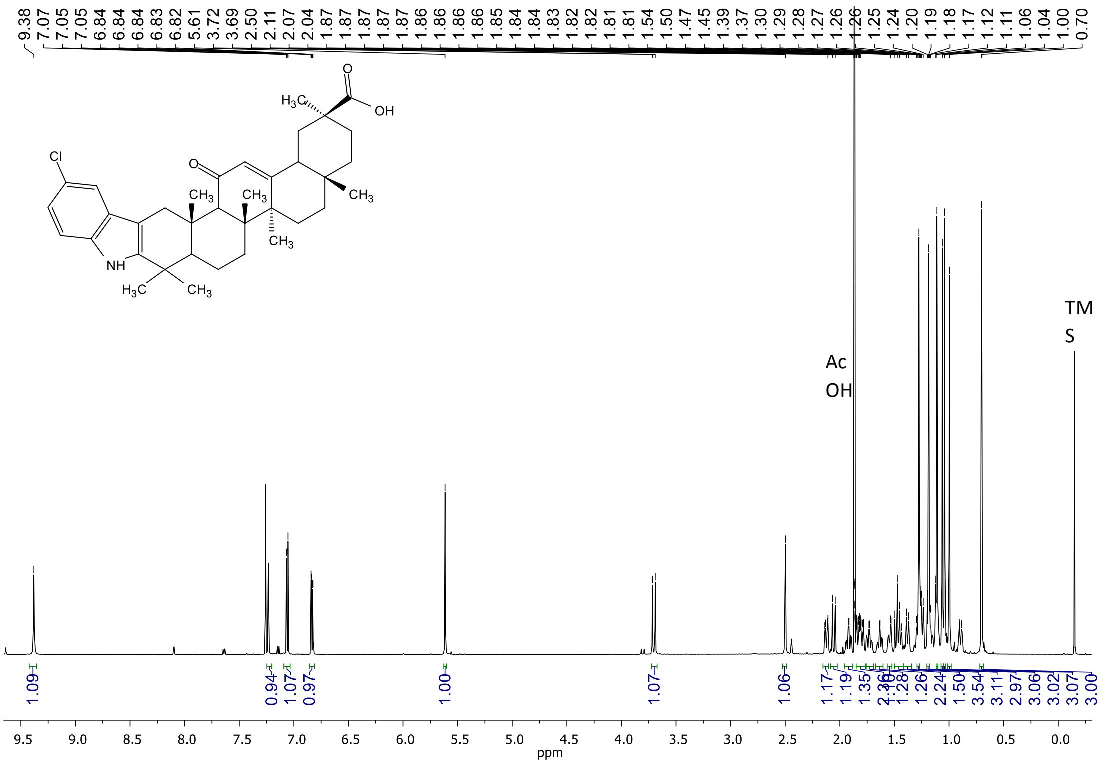

Figure S9. ^{1}H NMR (600 MHz) spectrum of compound **4e** in $CDCl_3$

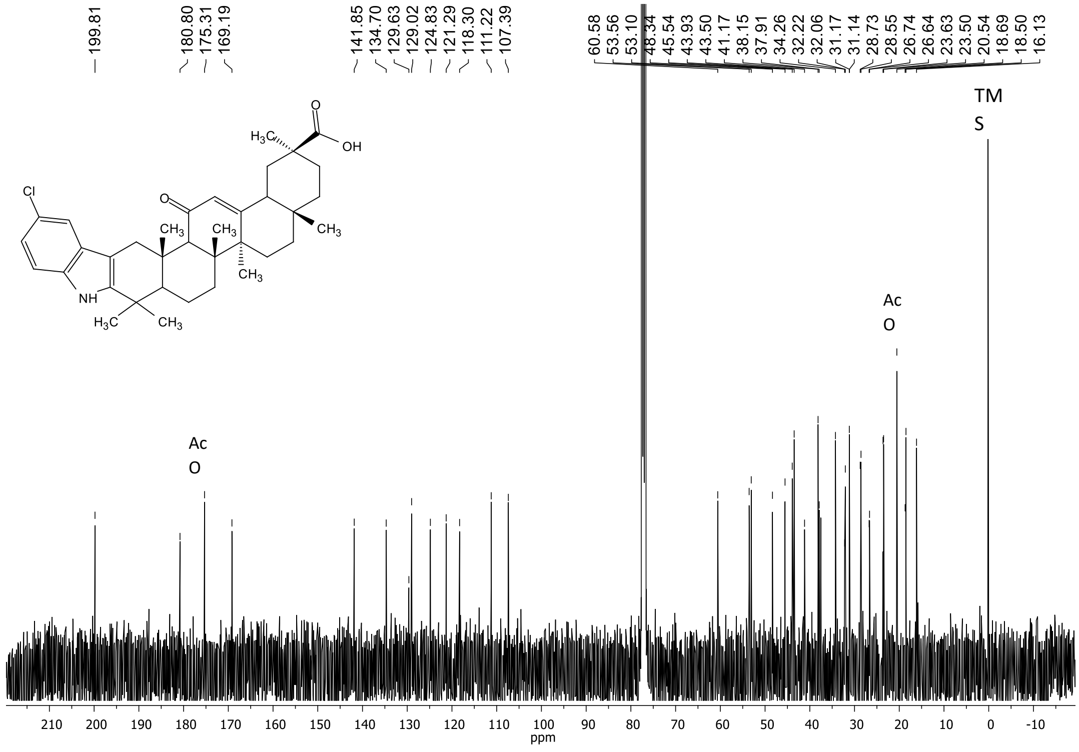

Figure S10. ^{13}C NMR (151 MHz) spectrum of compound **4e** in $CDCl_3$

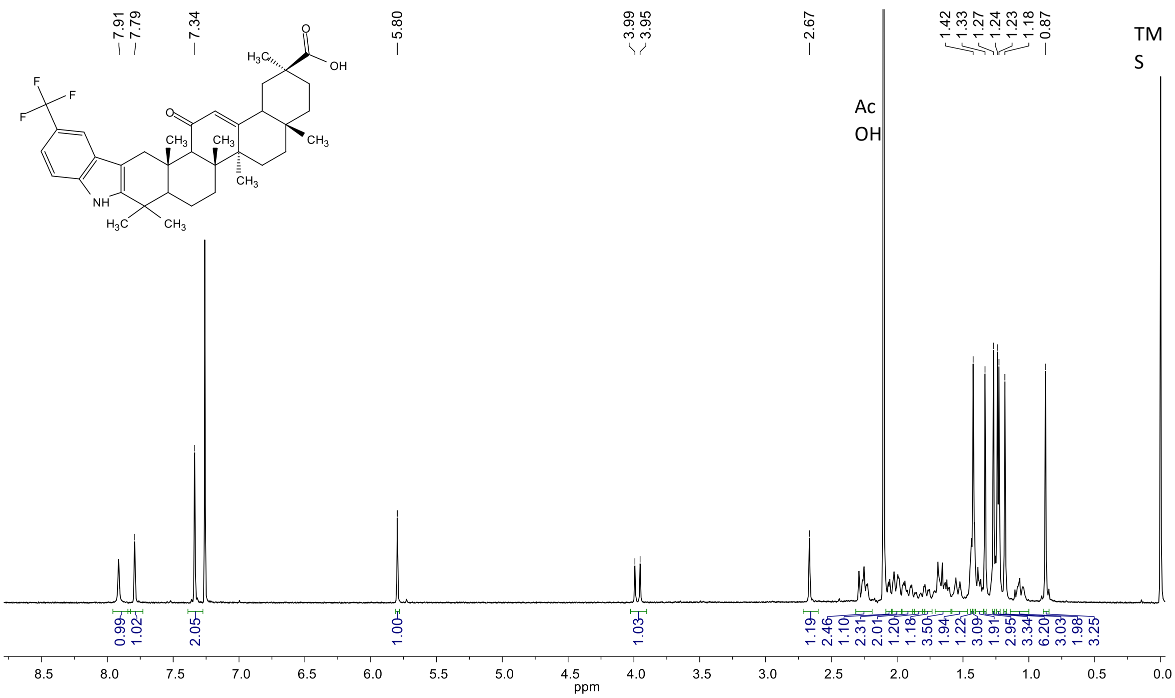

Figure S11. ^{1}H NMR (400 MHz) spectrum of compound **4f** in $CDCl_3$

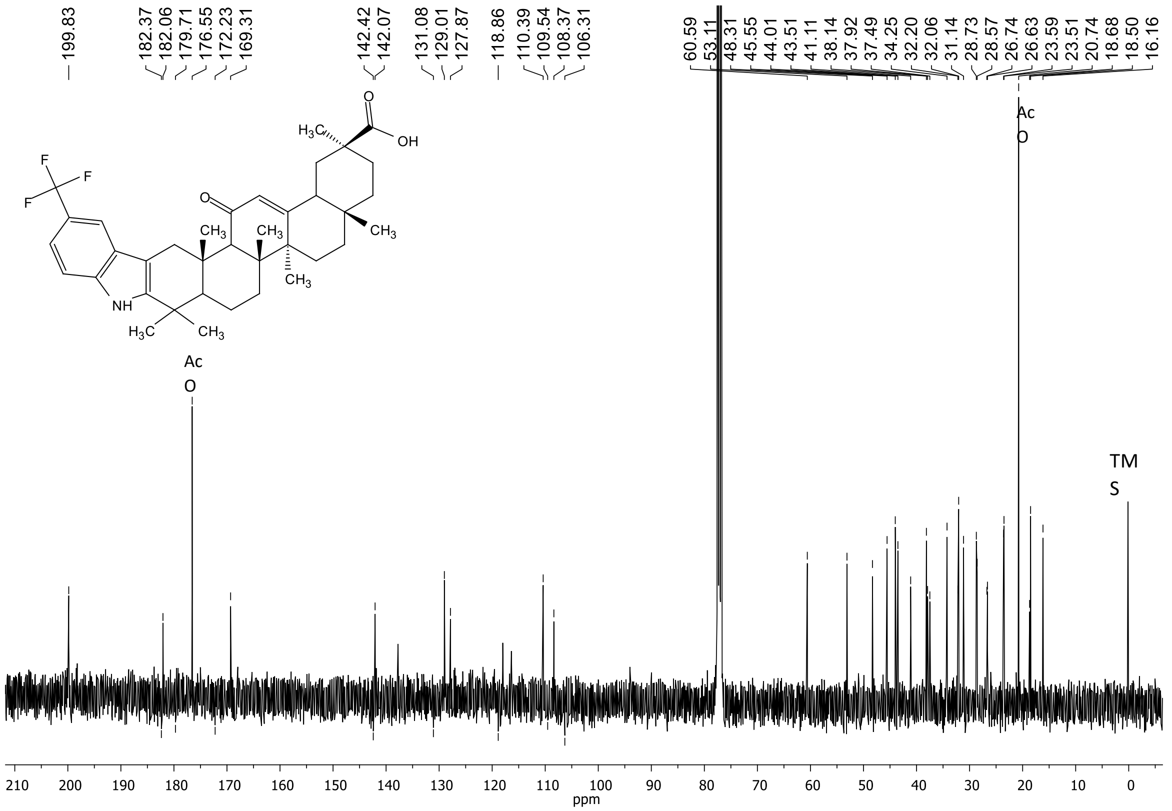

Figure S12. ^{13}C NMR (151 MHz) spectrum of compound **4f** in $CDCl_3$

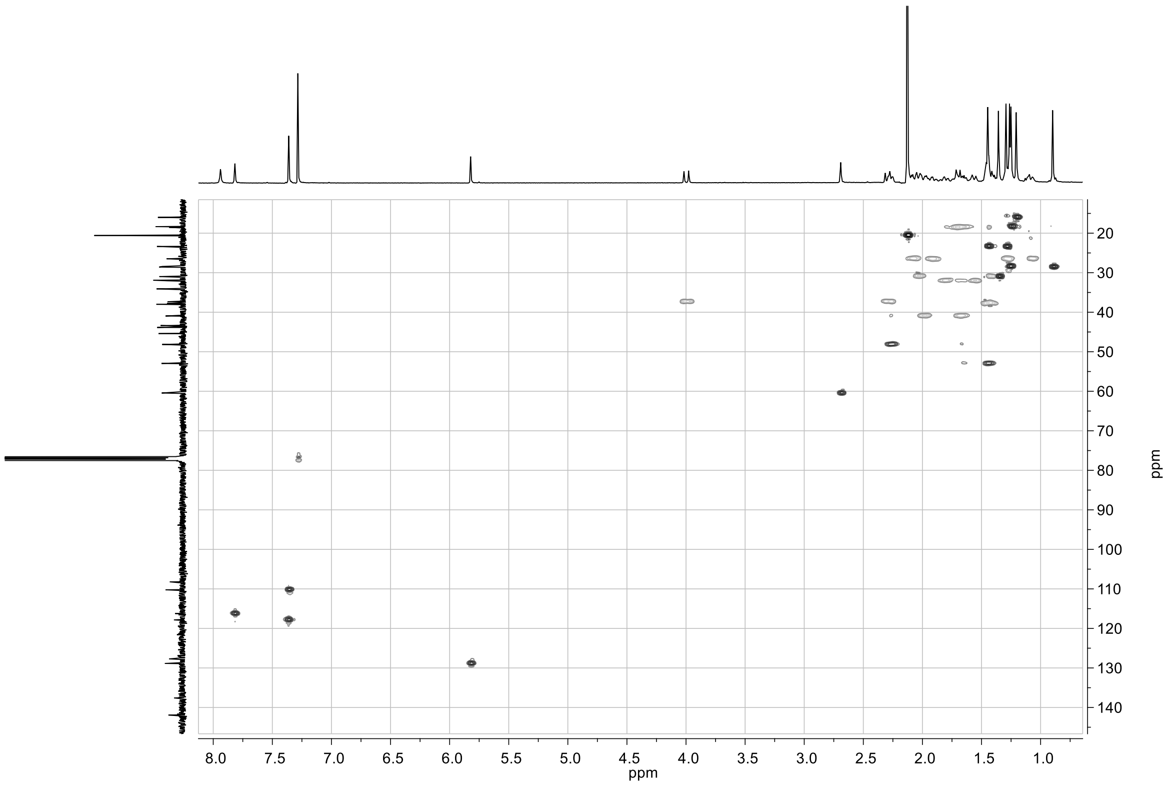

Figure S13. HSQC spectrum of compound **4f** in $CDCl_3$

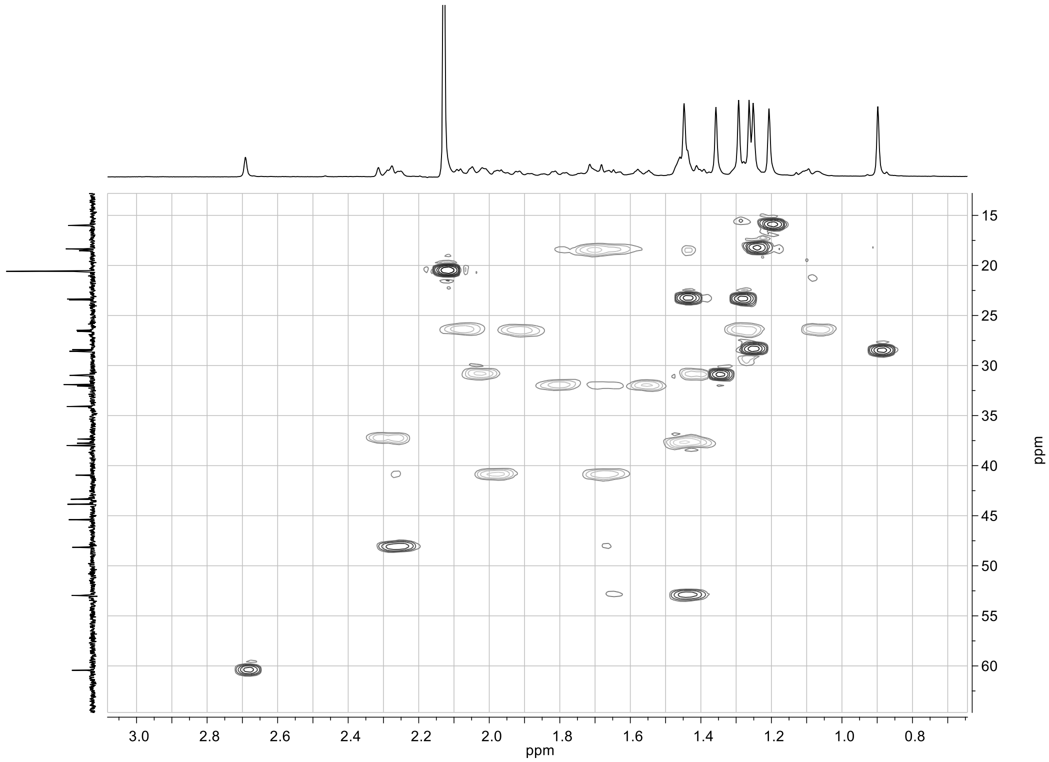

Figure S14. HSQC spectrum (0 – 3.0 ppm) of compound **4f** in $CDCl_3$

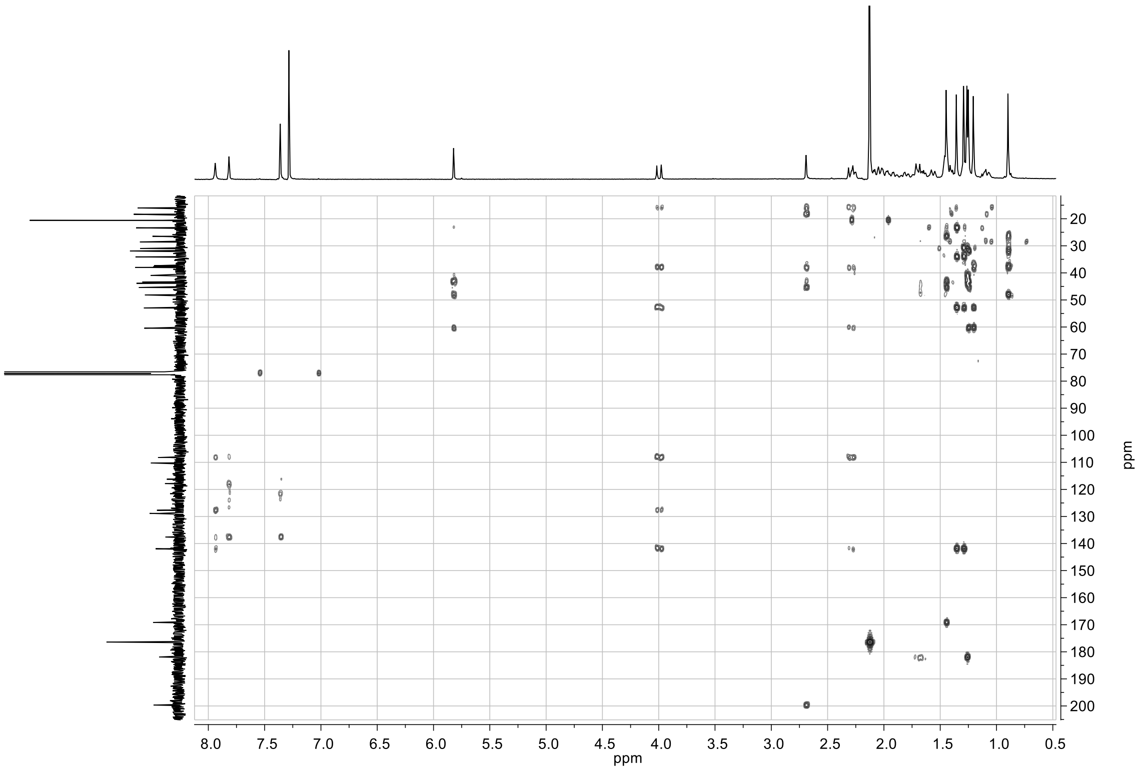

Figure S15. HMBC spectrum of compound **4f** in $CDCl_3$

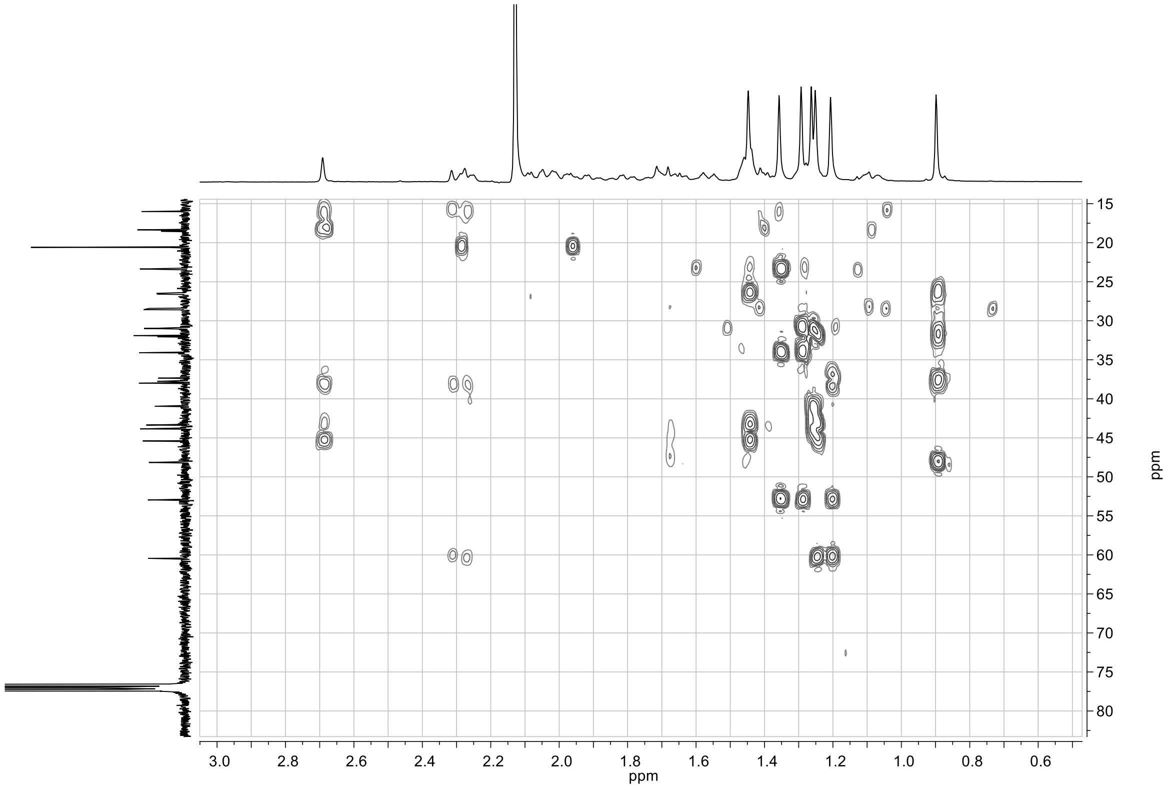

Figure S16. HMBC spectrum (0 – 3.0 ppm) of compound **4f** in $CDCl_3$

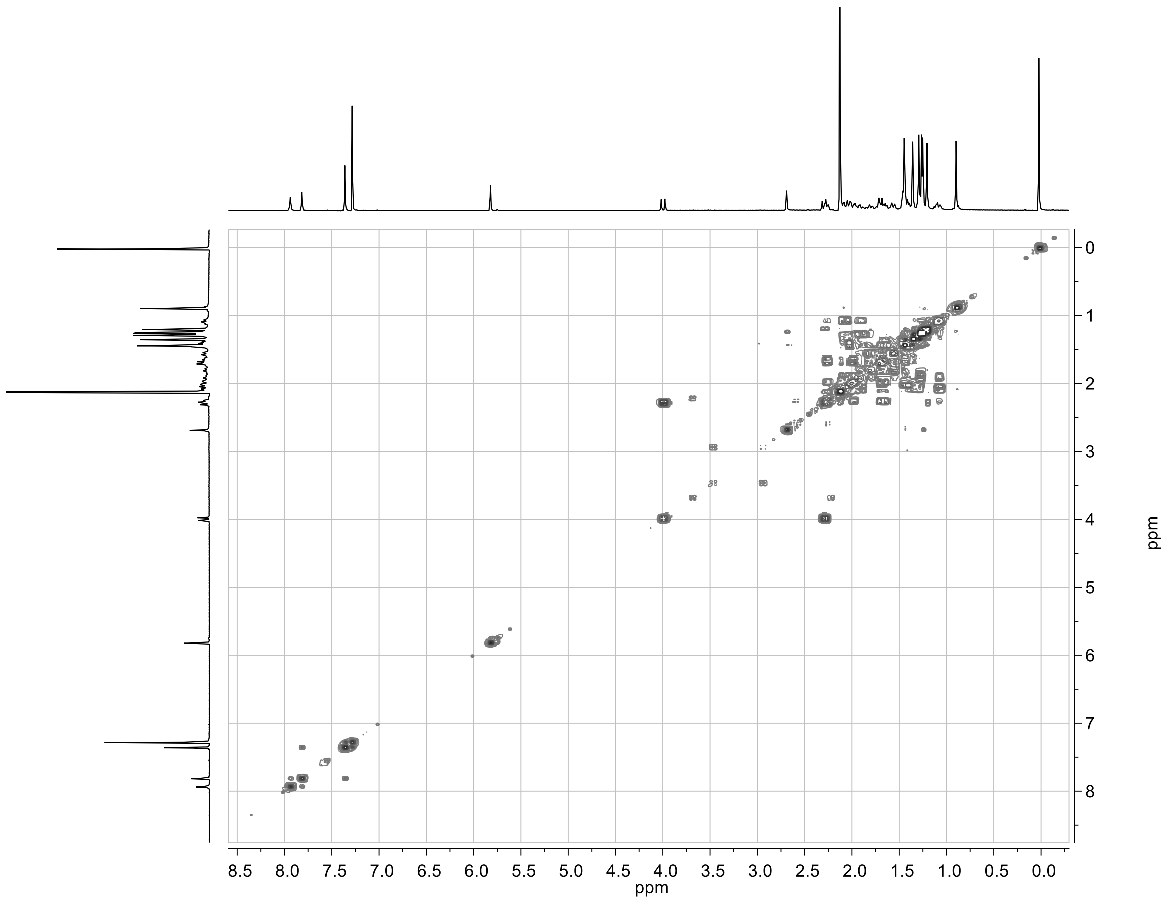

Figure S17. COSY spectrum of compound **4f** in $CDCl_3$

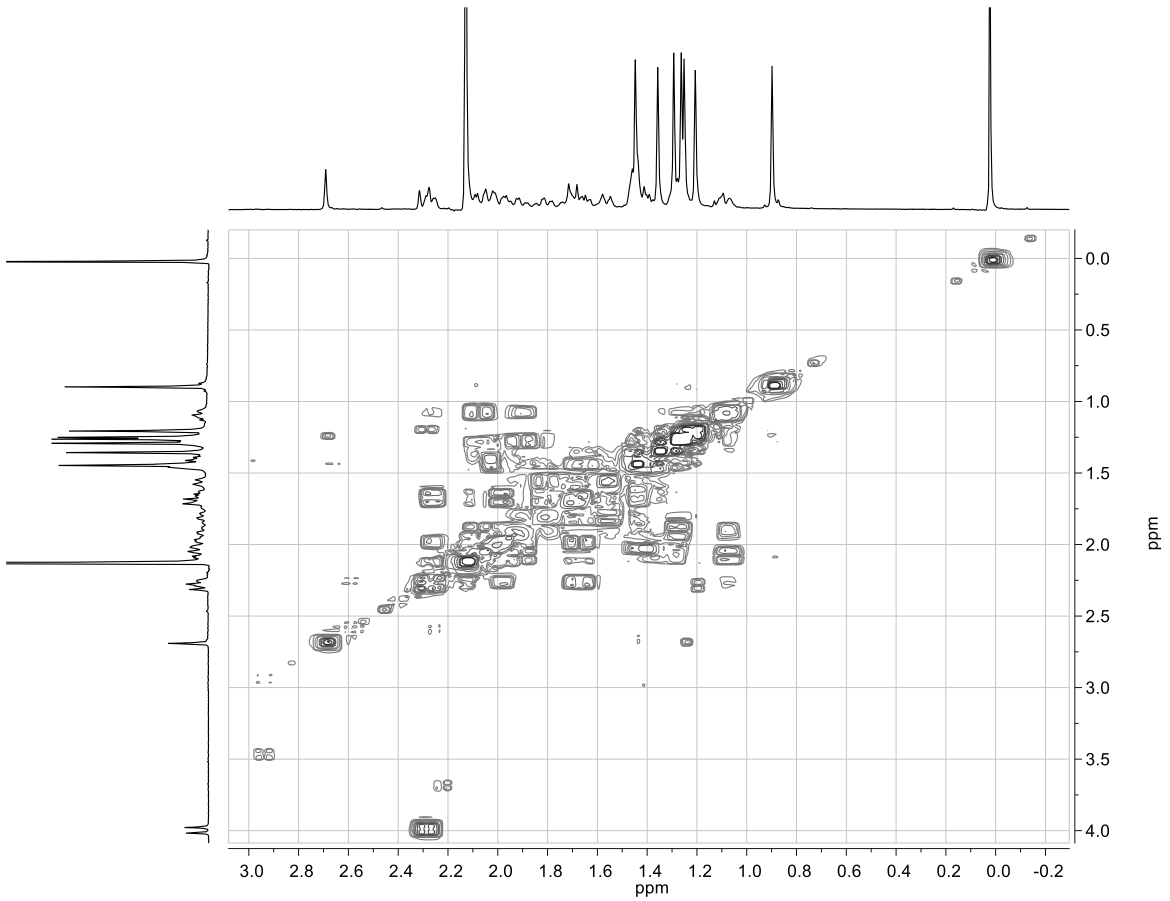

Figure S18. COSY spectrum (0 – 3.0 ppm) of compound **4f** in $CDCl_3$

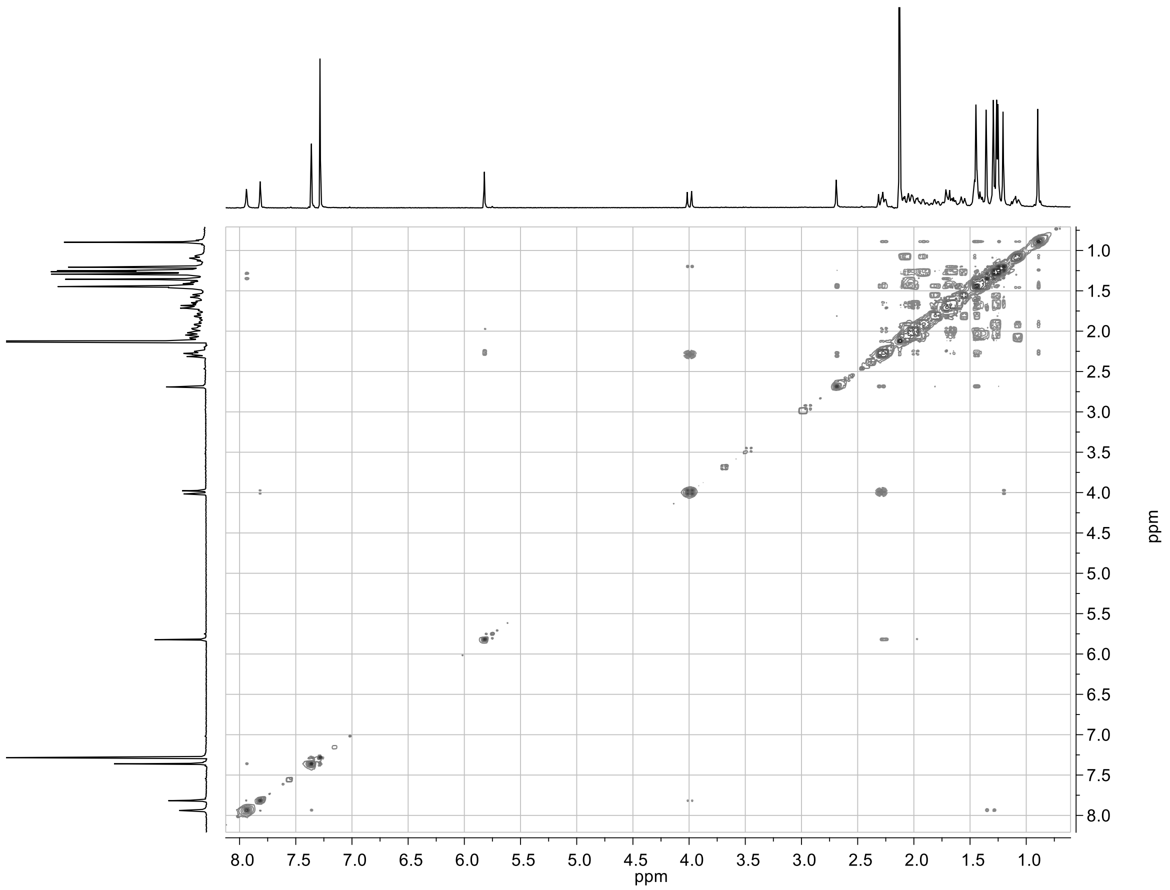

Figure S19. NOESY spectrum of compound **4f** in $CDCl_3$

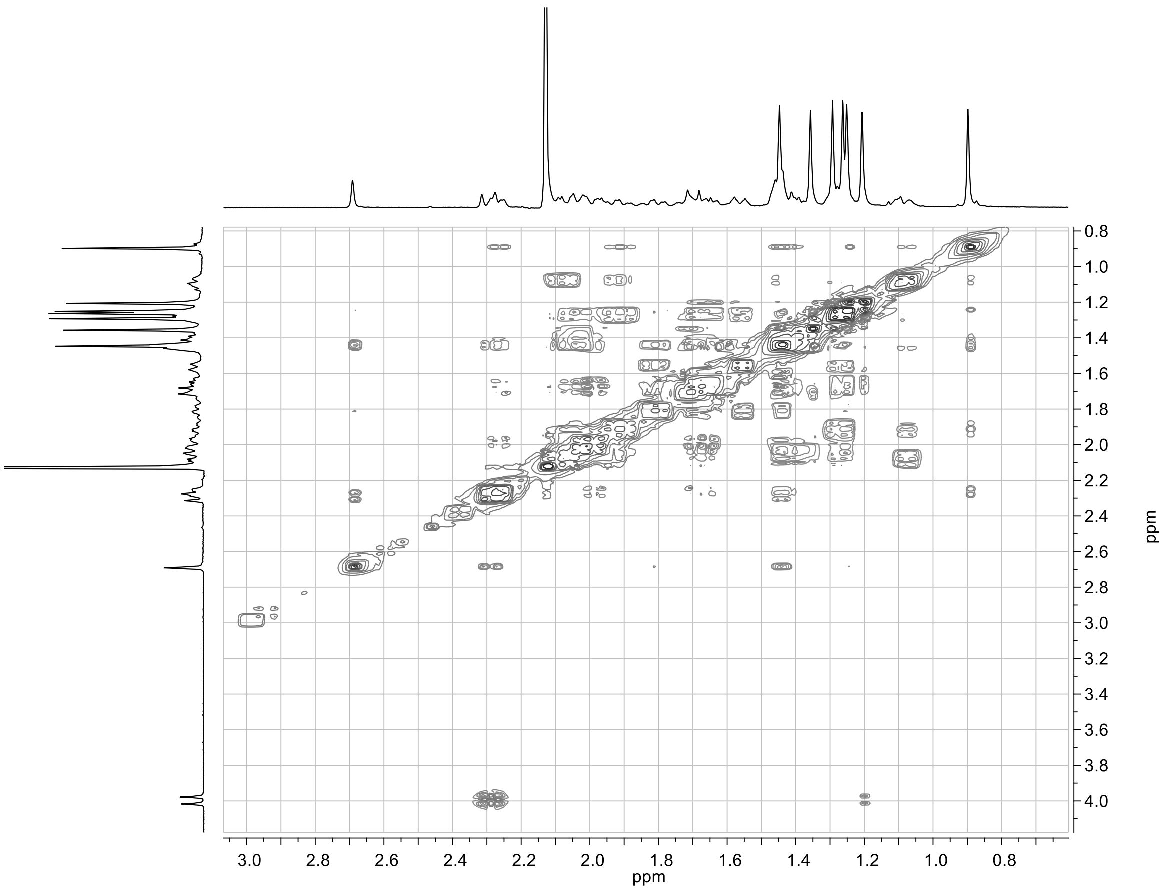

Figure S20. NOESY spectrum (0 – 3.0 ppm) of compound **4f** in $CDCl_3$

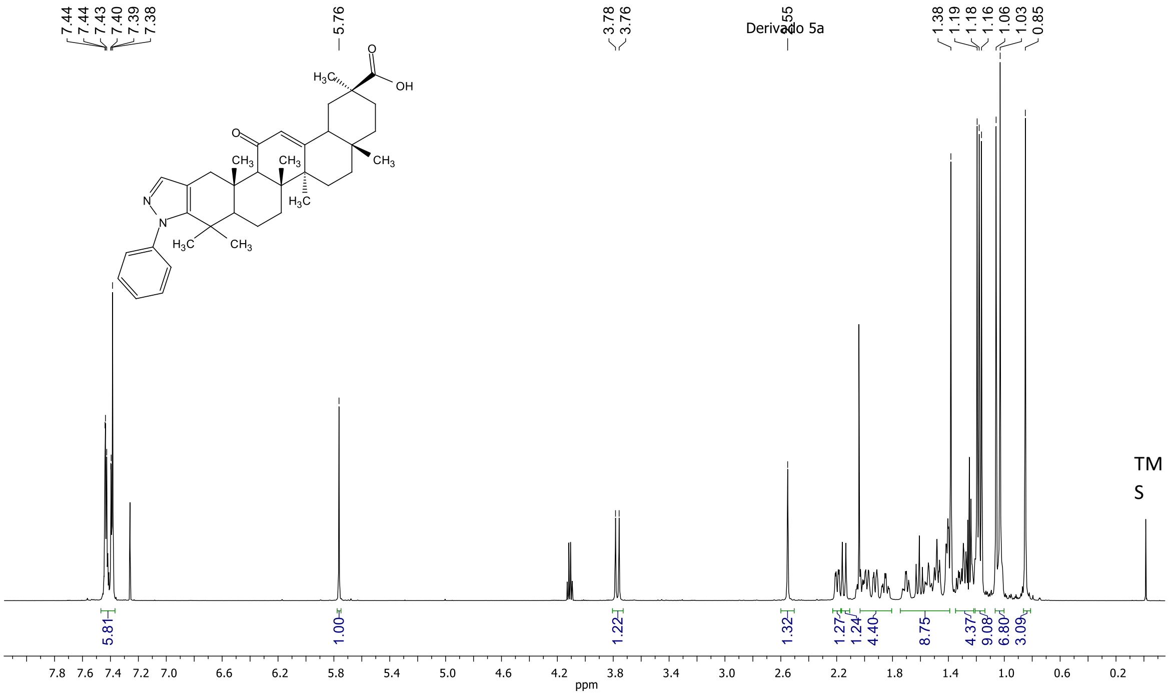

Figure S21. ^{1}H NMR (600 MHz) spectrum of compound **5a** in $CDCl_3$

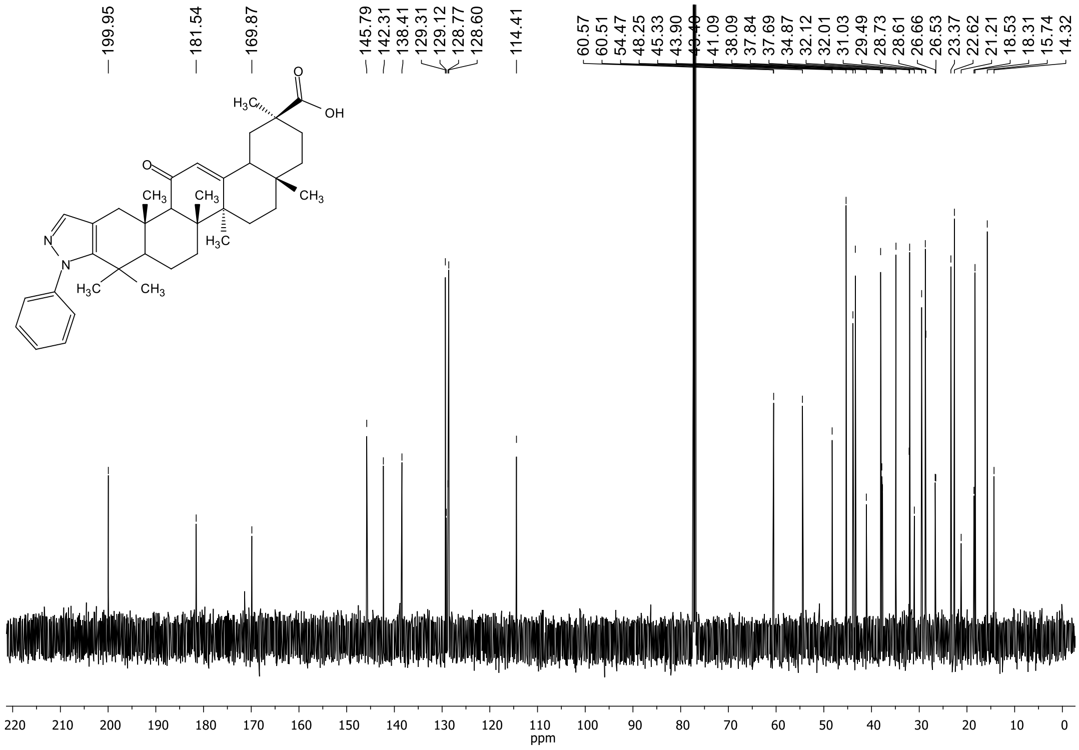

Figure S22. ^{13}C NMR (151 MHz) spectrum of compound **5a** in $CDCl_3$

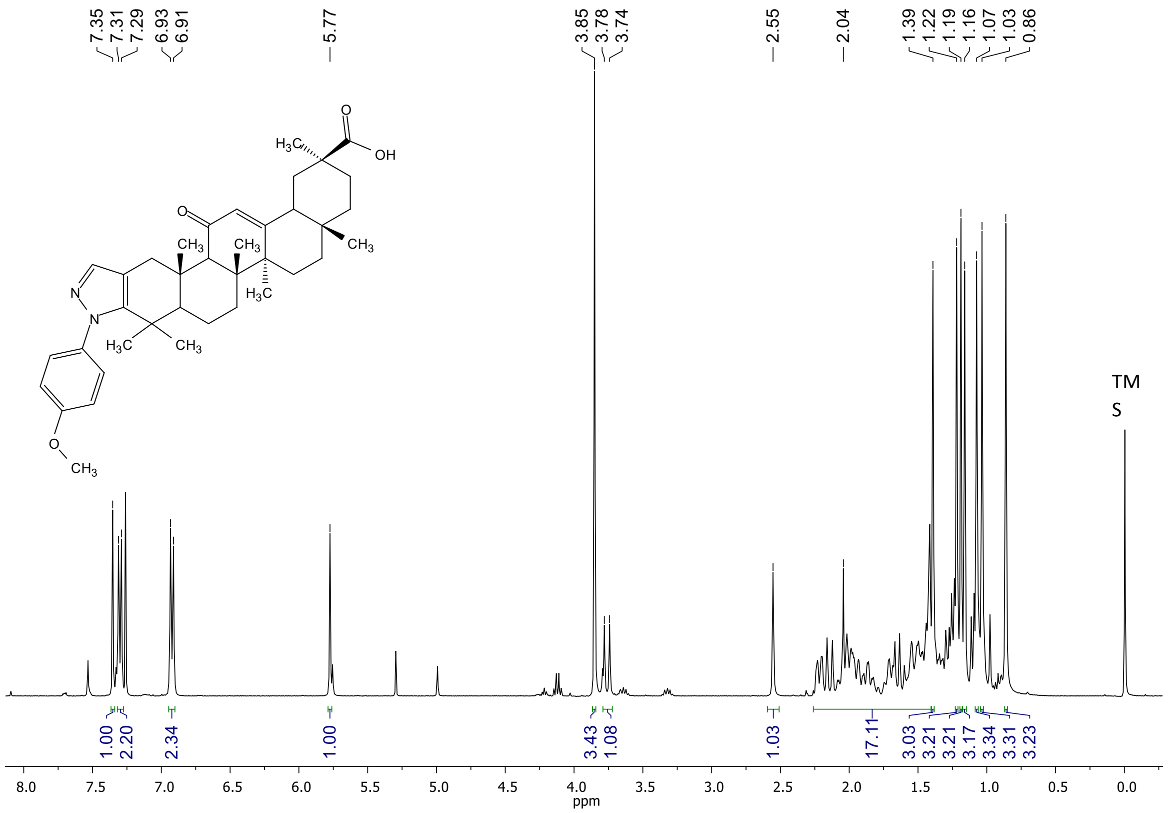

Figure S23. ^{1}H NMR (400 MHz) spectrum of compound **5b** in $CDCl_3$

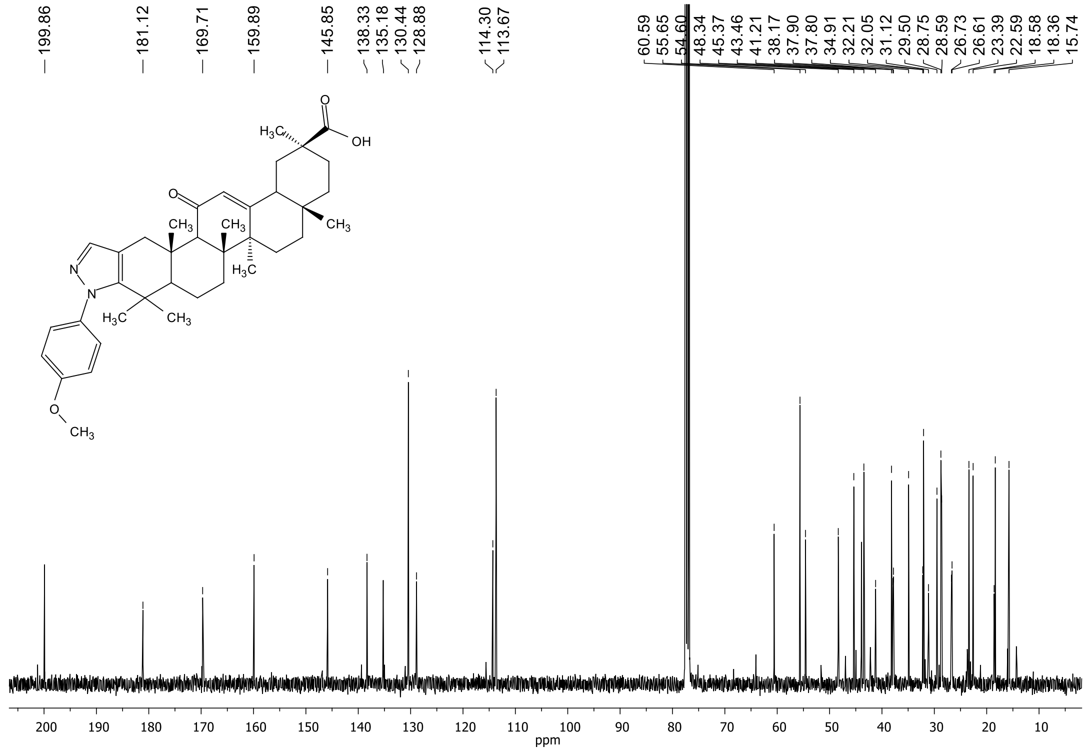

Figure S24. ^{13}C NMR (151 MHz) spectrum of compound **5b** in $CDCl_3$

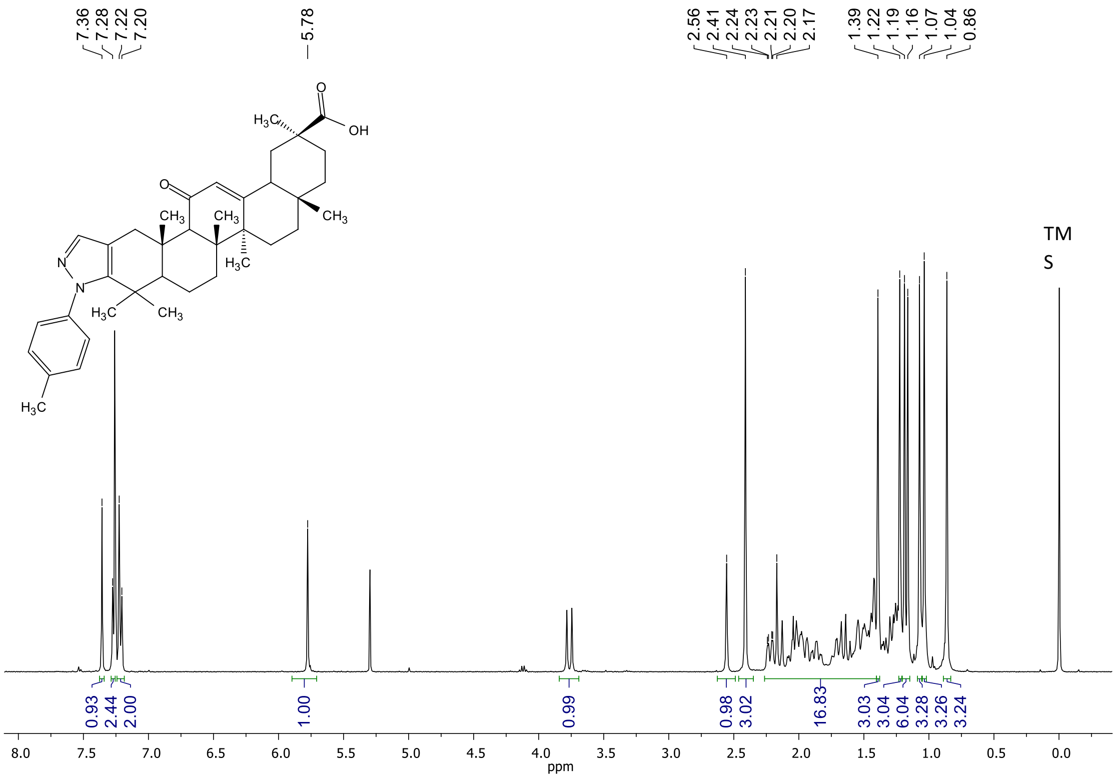

Figure S25. ^{1}H NMR (400 MHz) spectrum of compound **5c** in $CDCl_3$

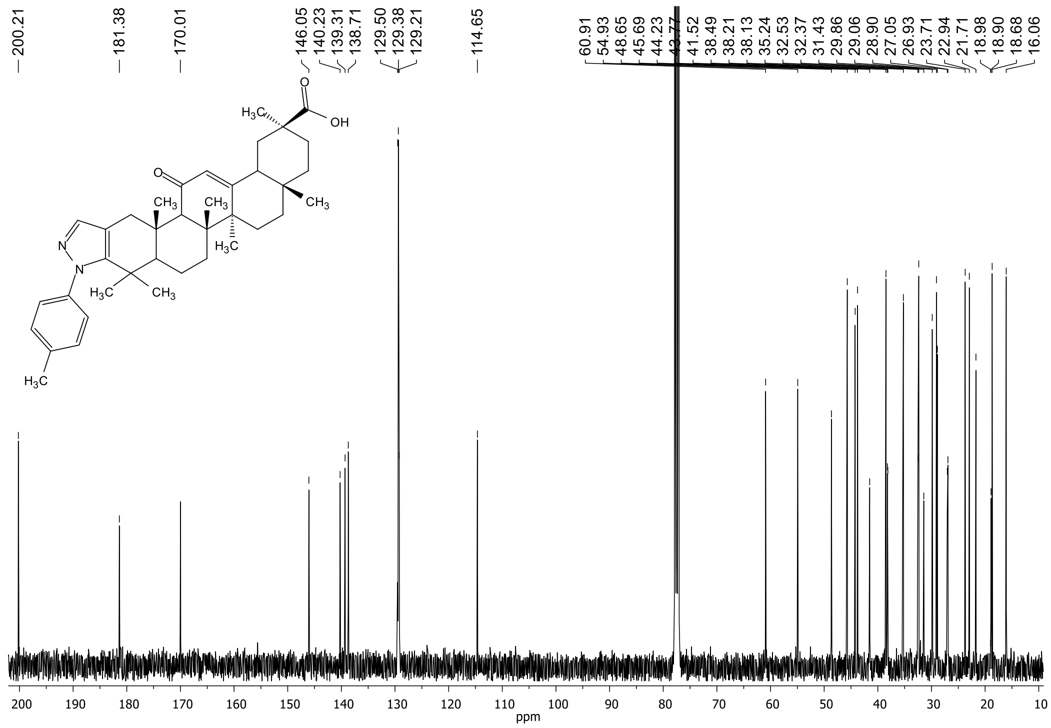

Figure S26. ^{13}C NMR (151 MHz) spectrum of compound **5c** in $CDCl_3$

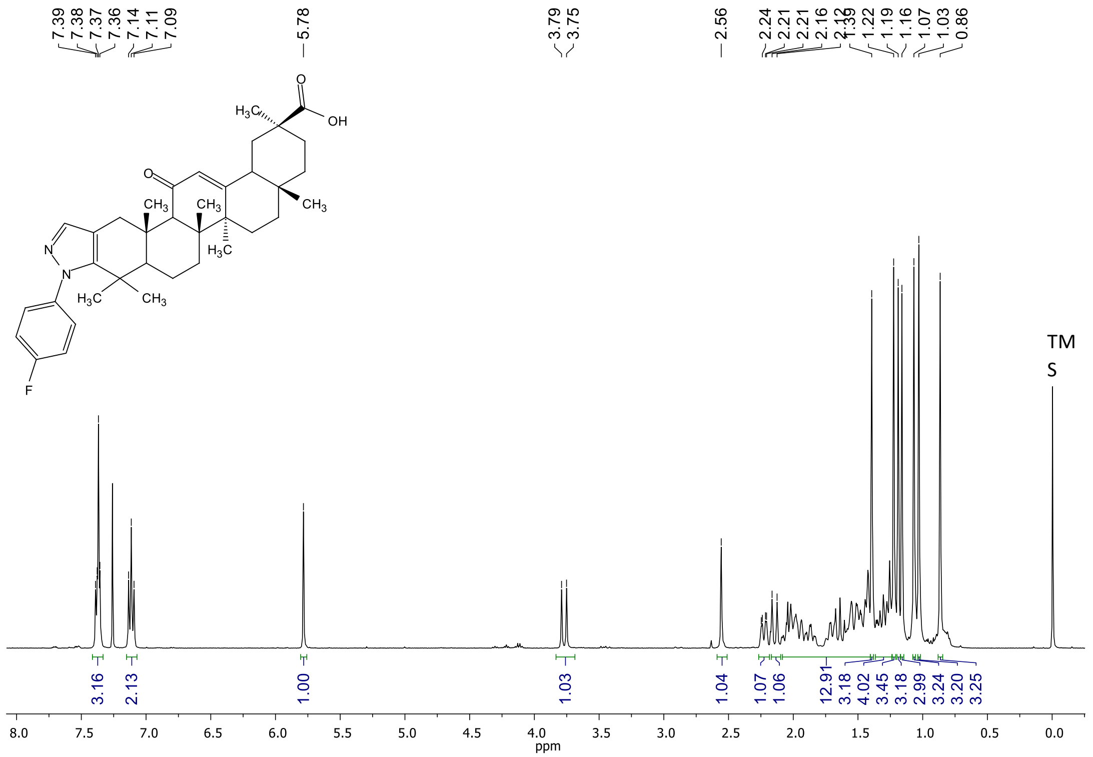

Figure S27. ^{1}H NMR (400 MHz) spectrum of compound **5d** in $CDCl_3$

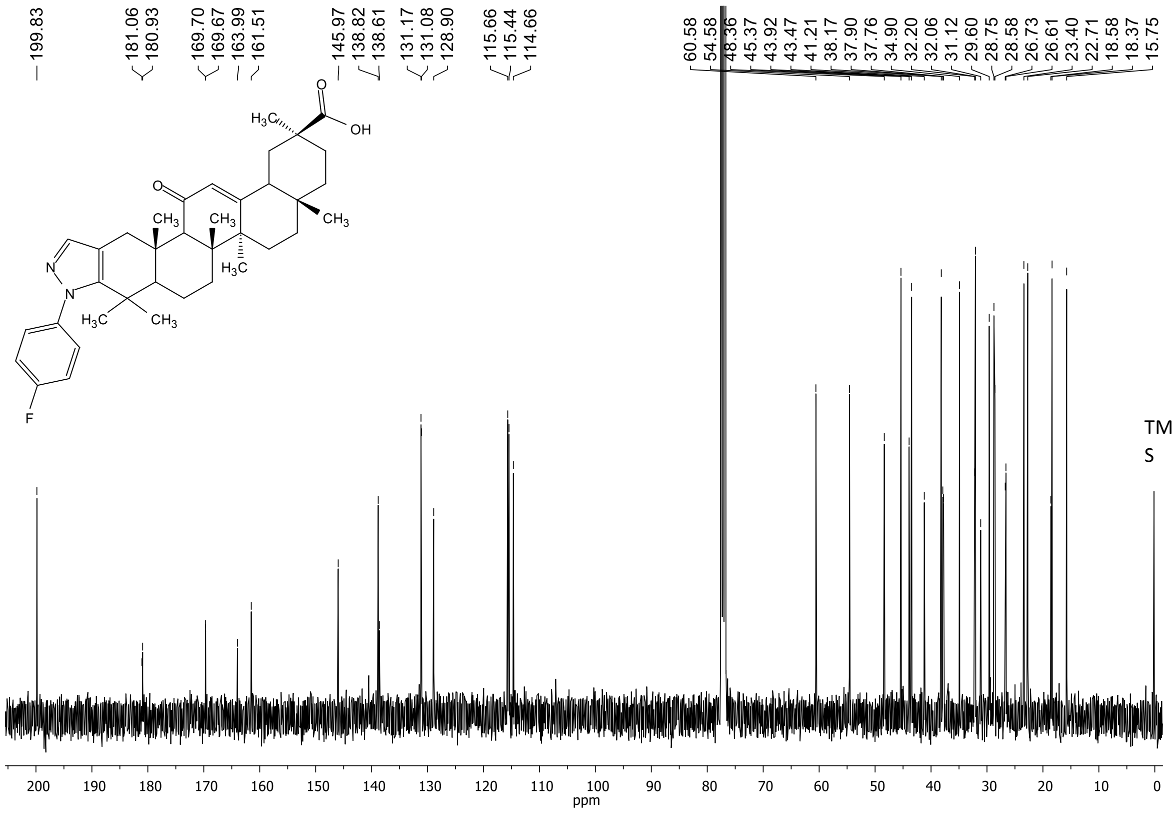

Figure S28. ^{13}C NMR (151 MHz) spectrum of compound **5d** in $CDCl_3$

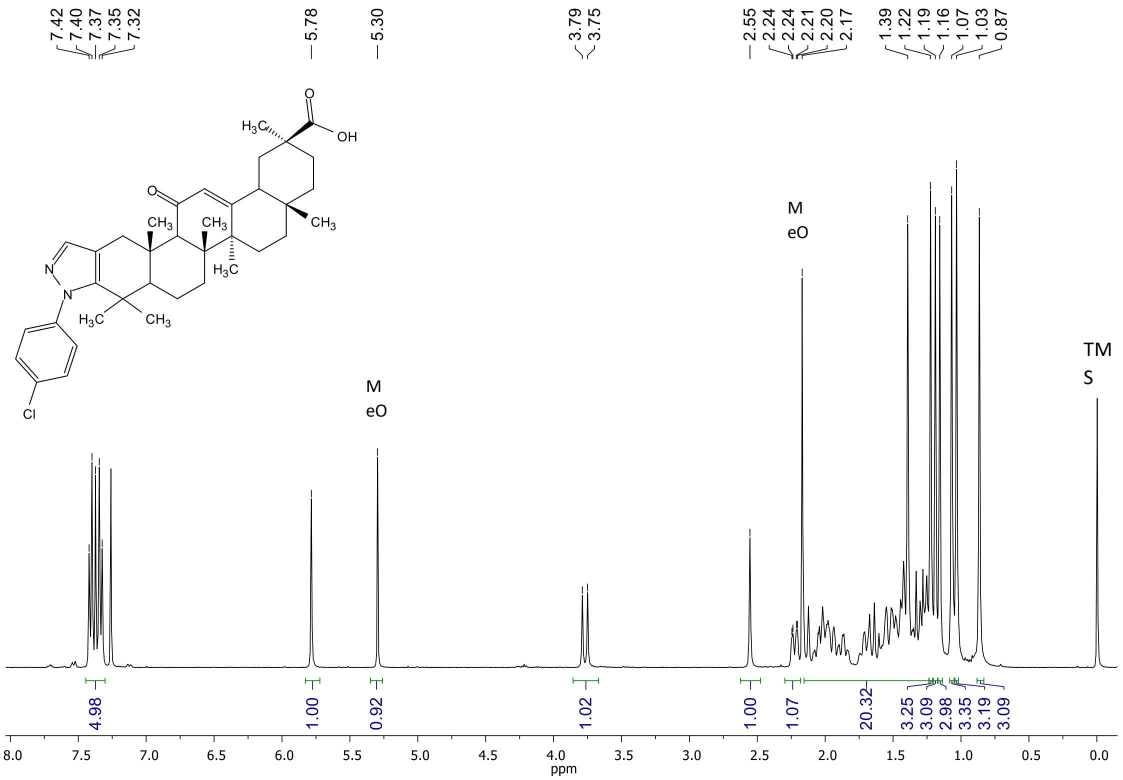

Figure S29. ^{1}H NMR (400 MHz) spectrum of compound **5f** in $CDCl_3$

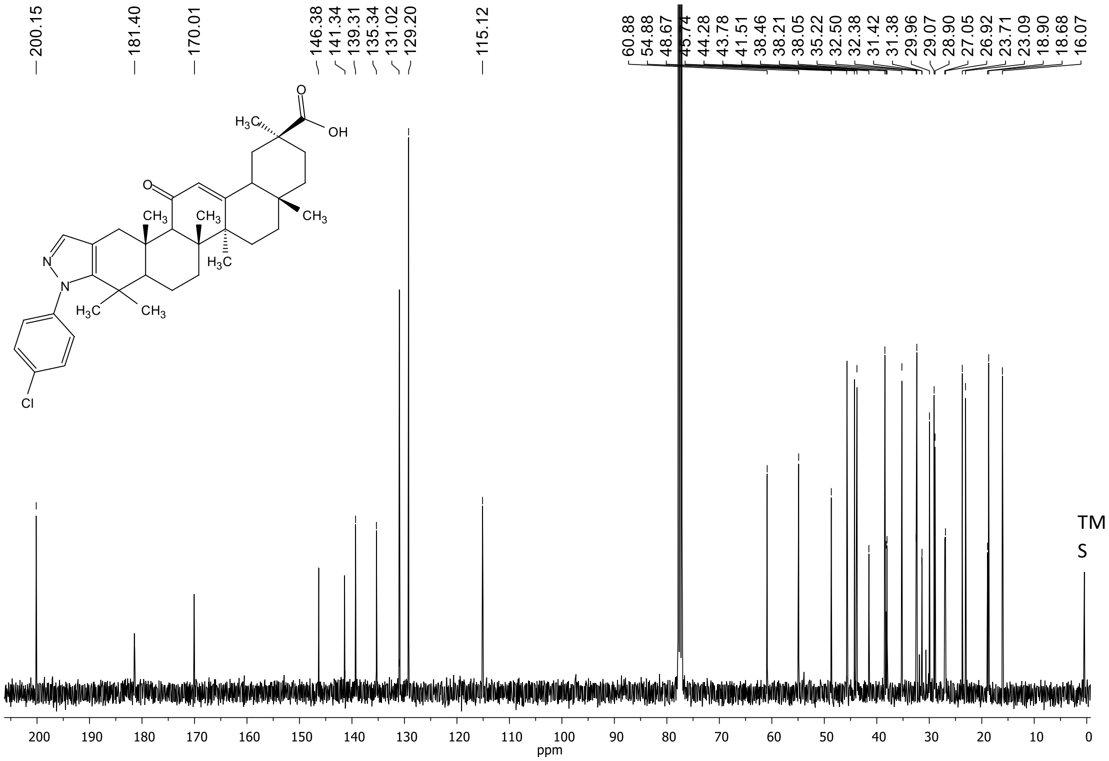

Figure S30. ^{13}C NMR (151 MHz) spectrum of compound **5f** in $CDCl_3$

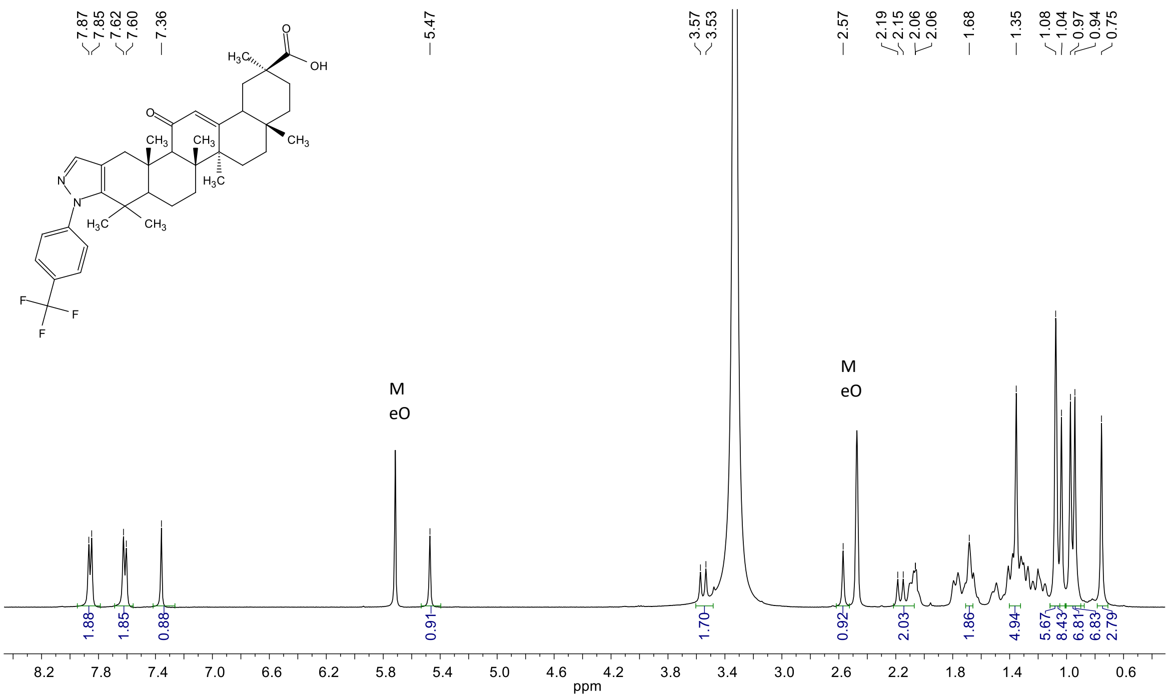

Figure S31. ^{1}H NMR (400 MHz) spectrum of compound **5f** in DMSO-d6

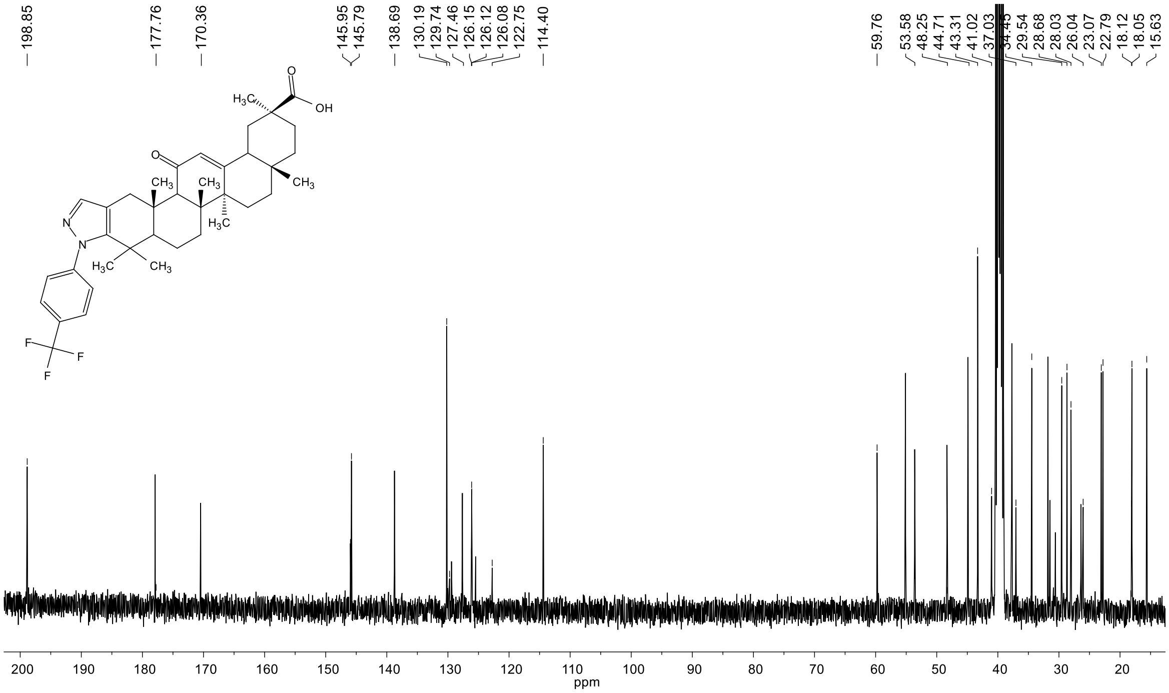

Figure S32. ^{13}C NMR (151 MHz) spectrum of compound **5f** in DMSO-d6

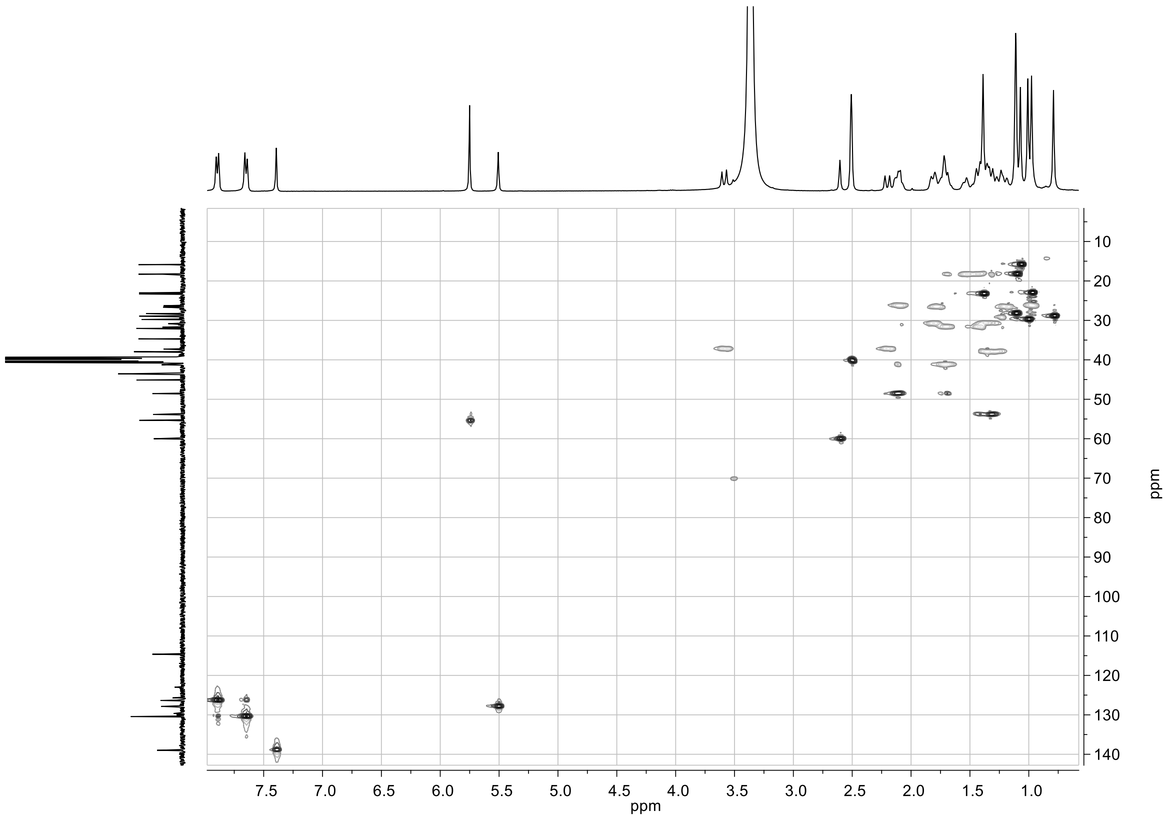

Figure S33. HSQC spectrum of compound **5f** in DMSO-d_6

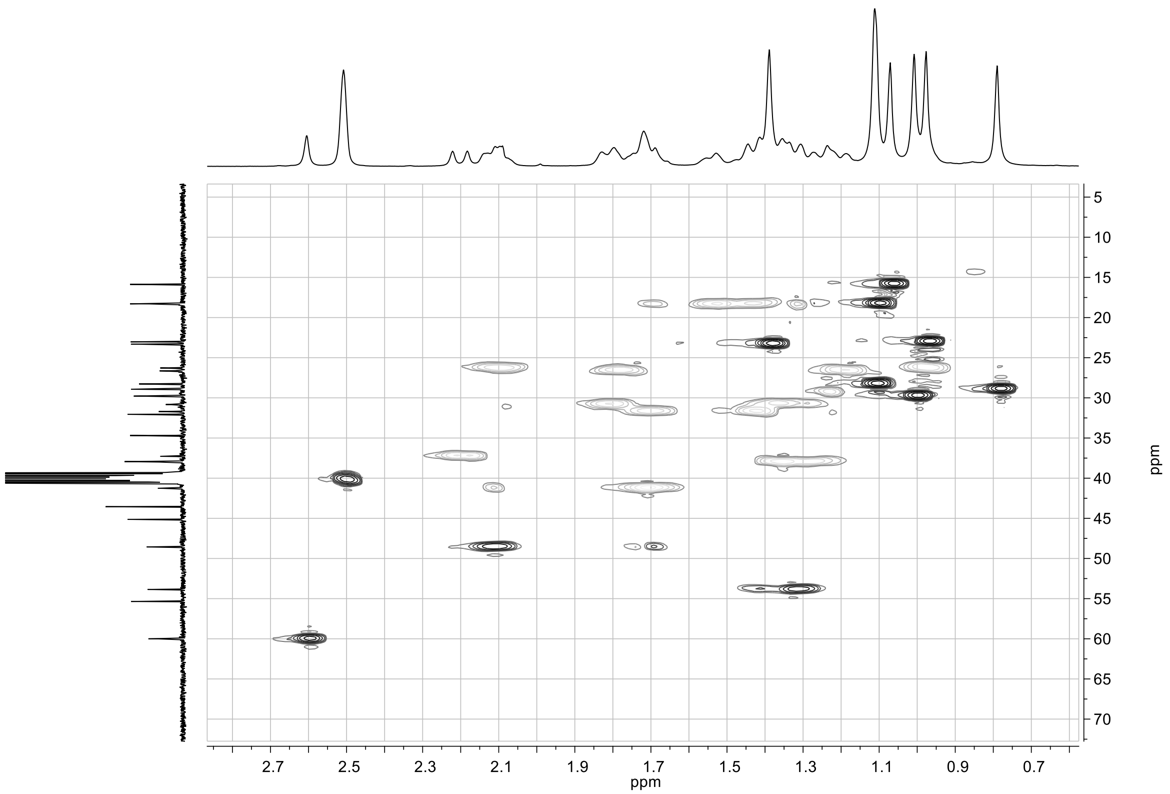

Figure S34. HSQC spectrum (0 – 2.7 ppm) of compound **5f** in DMSO-d_6

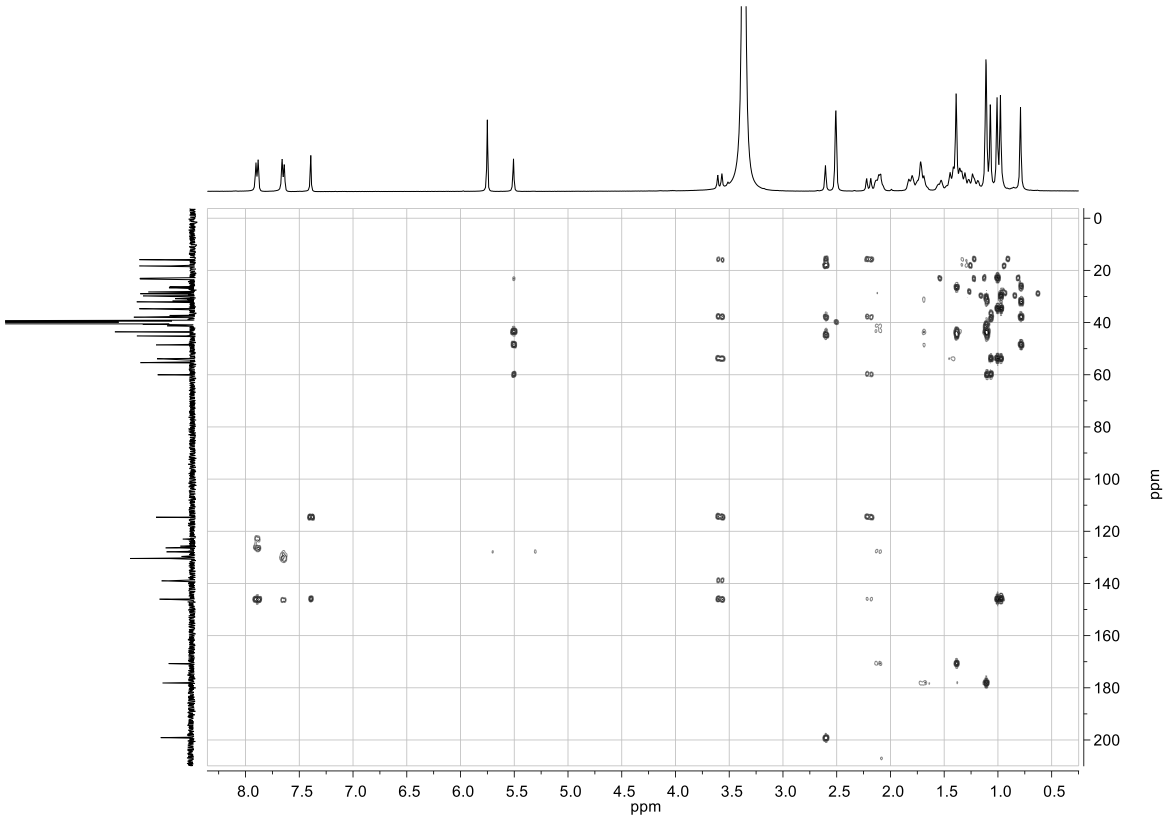

Figure S35. HMBC spectrum of compound **5f** in DMSO-d6

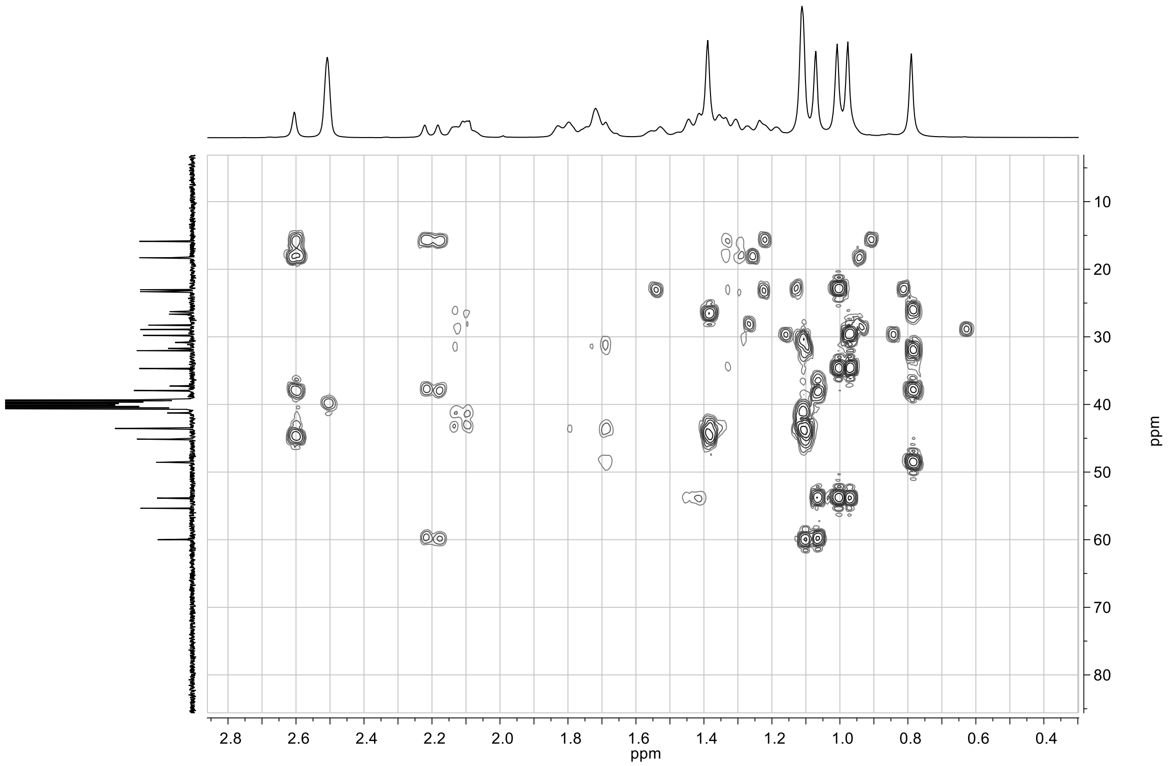

Figure S36. HMBC spectrum (0 – 2.8 ppm) of compound **5f** in DMSO-d6

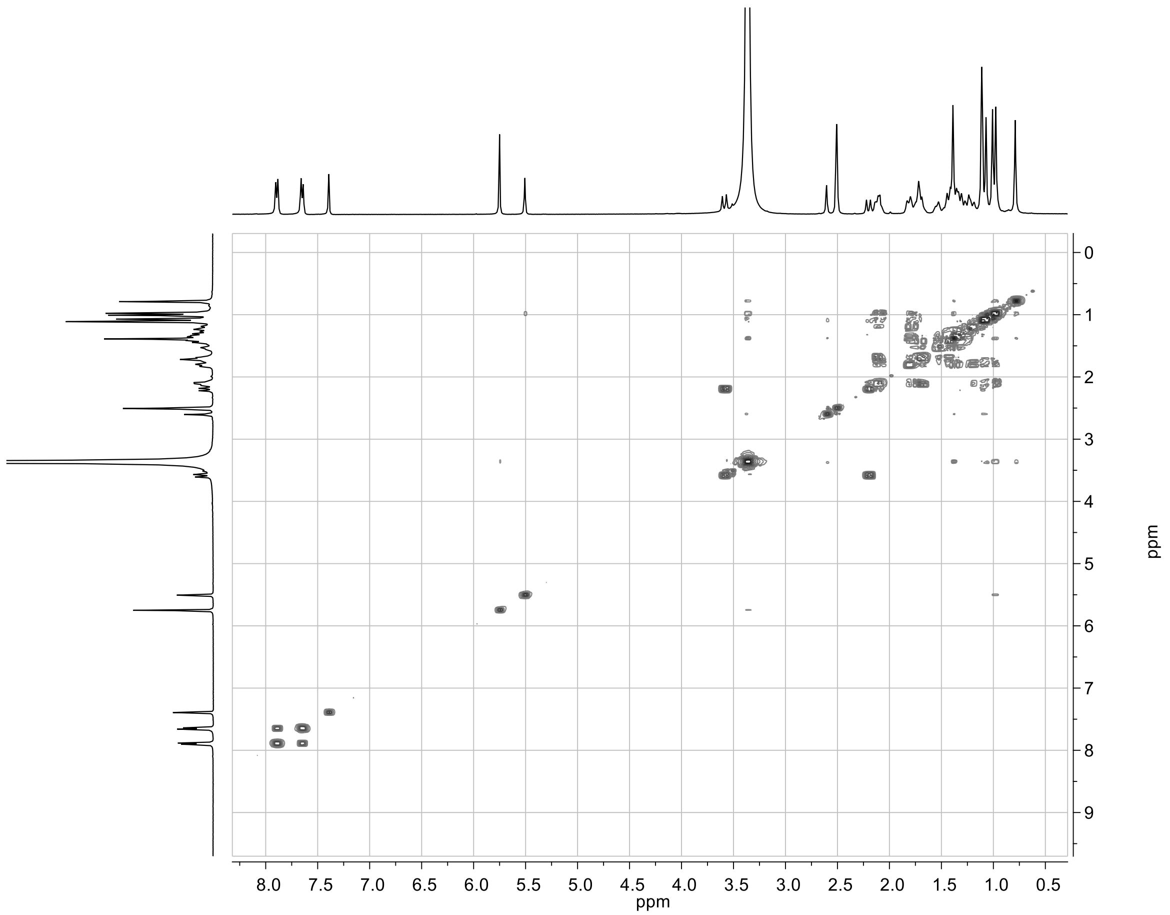

Figure S37. COSY spectrum of compound **5f** in DMSO-d_6

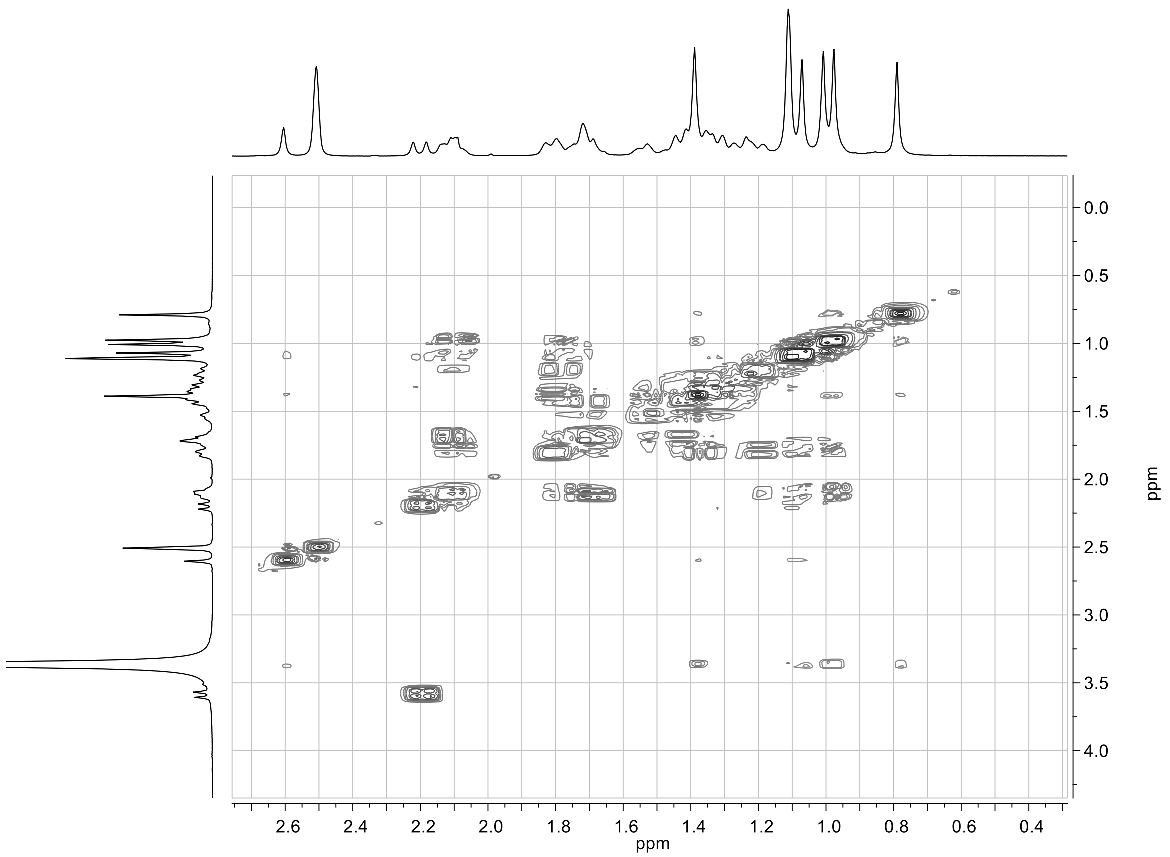

Figure S38. COSY spectrum (0 – 3.0 ppm) of compound **5f** in DMSO-d_6

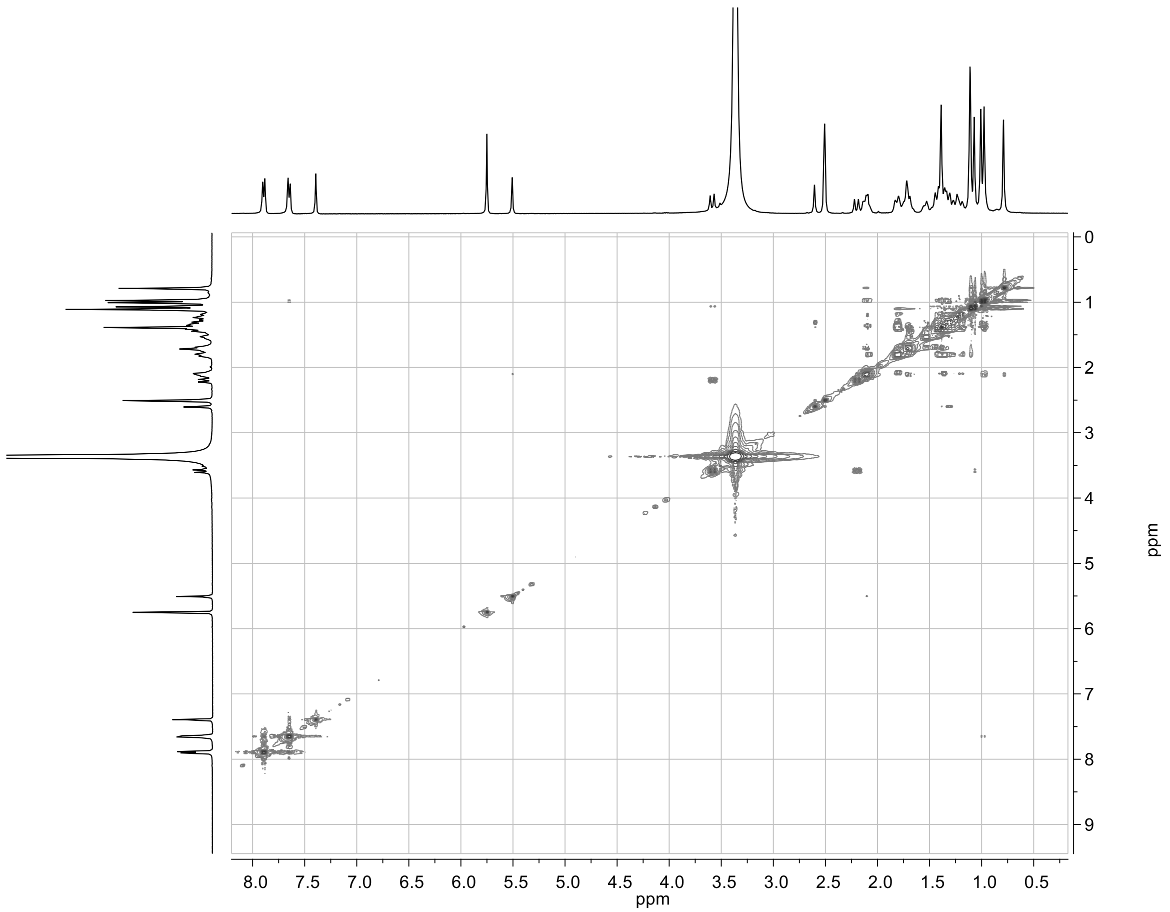

Figure S39. NOESY spectrum of compound **5f** in DMSO-d_6

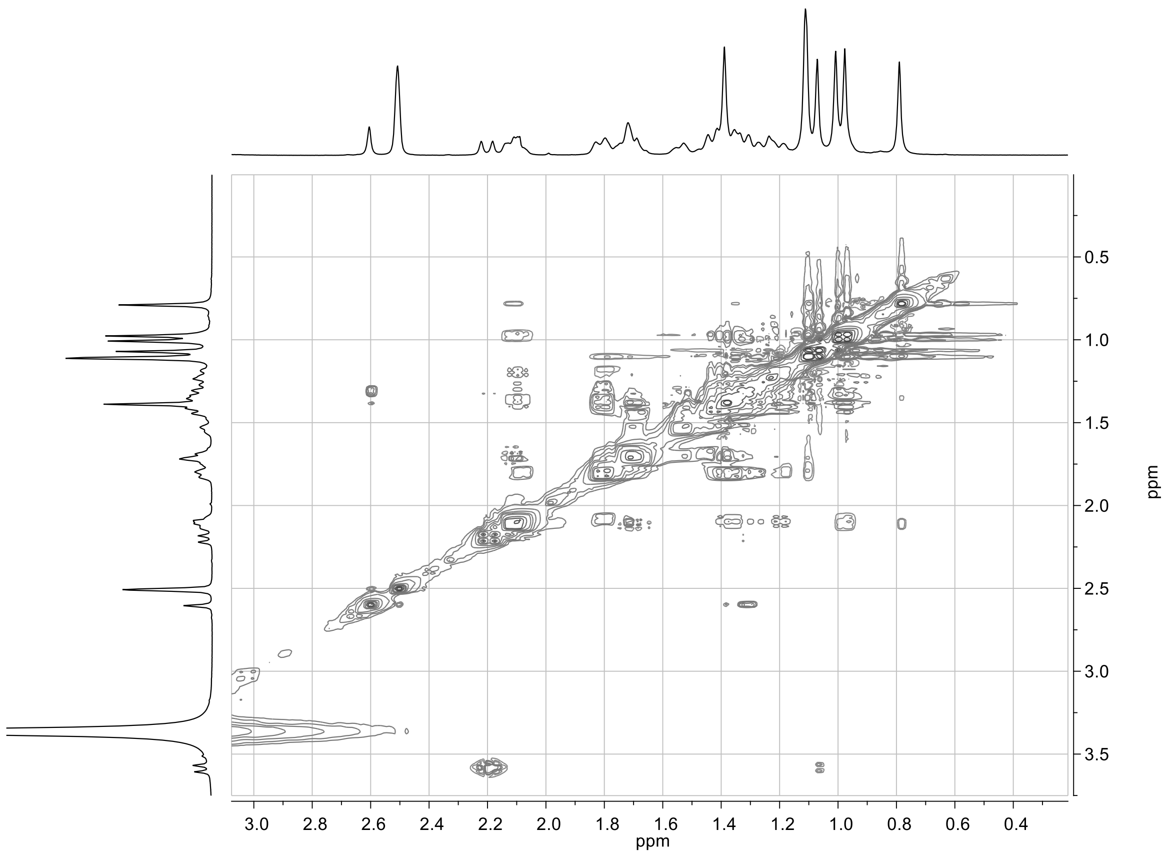

Figure S40. NOESY spectrum (0 – 3.0 ppm) of compound **5f** in DMSO-d_6

For the synthesis of indole-GA series, it is important to mention that we did not use *meta*-phenylhydrazines because we will obtain isomeric mixtures of these compounds which are difficult to purify.

SAMPLE INFORMATION

Sample Name:	FC-1-47	Acquired By:	
Sample Type:	Unknown	Sample Set Name	001 Prueba 17 FC 1 47 dil
Vial:	2:A,2	Acq. Method Set:	ID 001 QPDA
Injection #:	1	Injection Volume:	10.00 ul
Run Time:	7.0 Minutes	Date Acquired:	6/2/2021 11:41:27 AM CDT
Column Name:			

ACQUITY UPLC HSS T3 1.8um 3.0 x 100 mm

Auto-Scaled Chromatogram

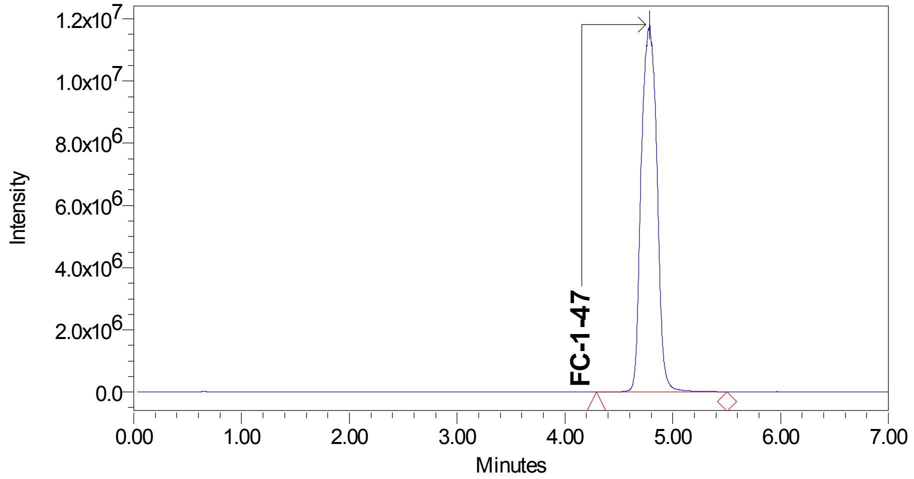

Peak Results

	Name	RT	Area	Height	MS 3D Channel Name
1	FC-1-47	4.786	119035283	11807244	QDa 1: MS Scan

Match Plot

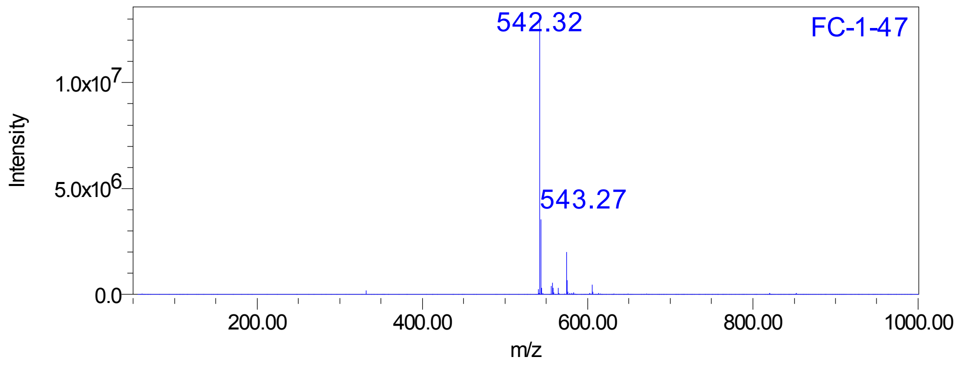

UPLC–ESI–MS data of compound 4a

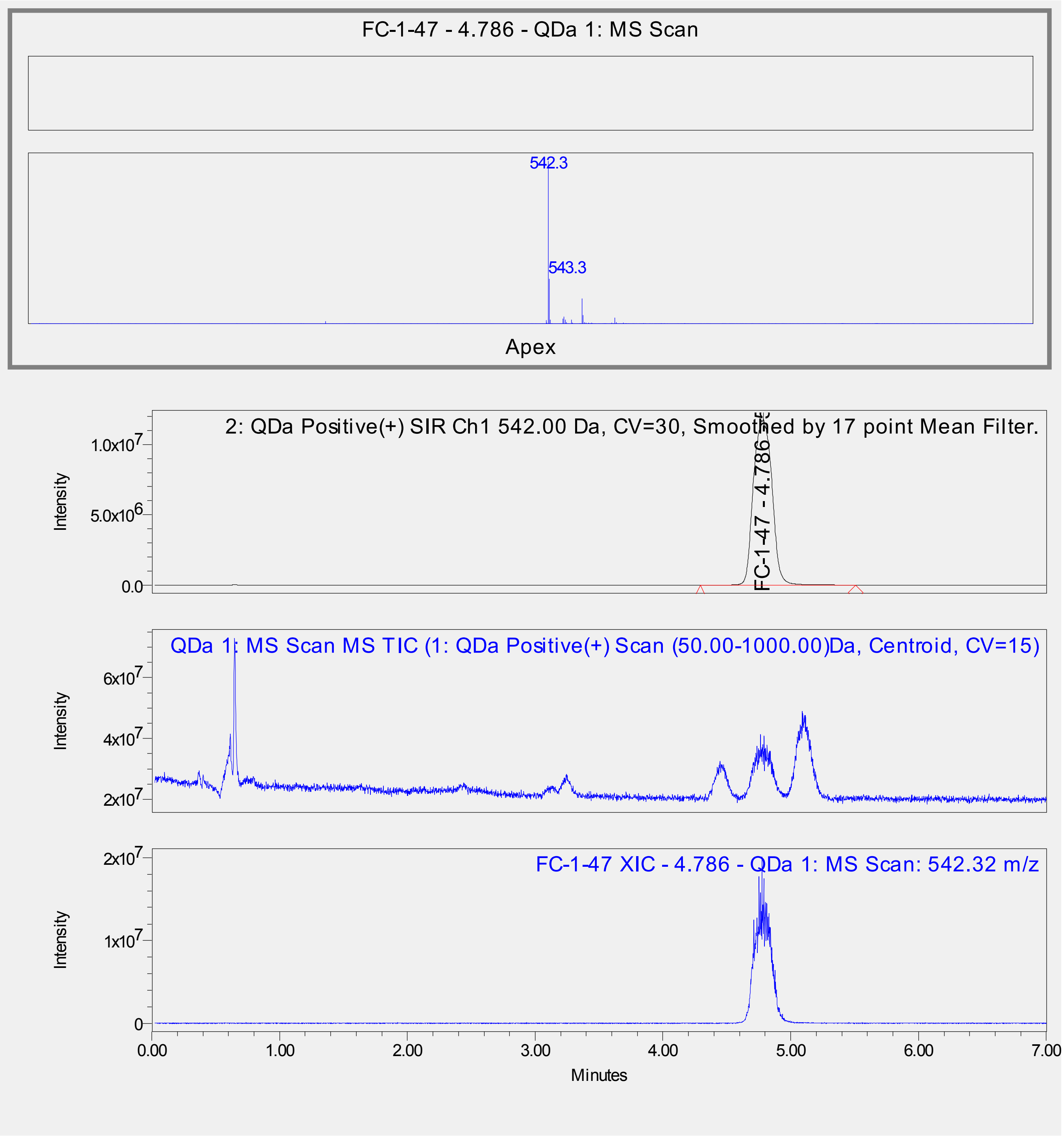

UPLC–ESI–MS data of compound 4a

SAMPLE INFORMATION

Sample Name:	CDB-2-24	Acquired By:	
Sample Type:	Unknown	Sample Set Name	001 Prueba 2 CDB 2 24 Dil
Vial:	2:B,3	Acq. Method Set:	ID 002 QPDA
Injection #:	1	Injection Volume:	10.00 ul
Run Time:	7.0 Minutes	Date Acquired:	6/2/2021 2:33:20 PM CDT
Column Name:			

ACQUITY UPLC HSS T3 1.8um 3.0 x 100 mm

Auto-Scaled Chromatogram

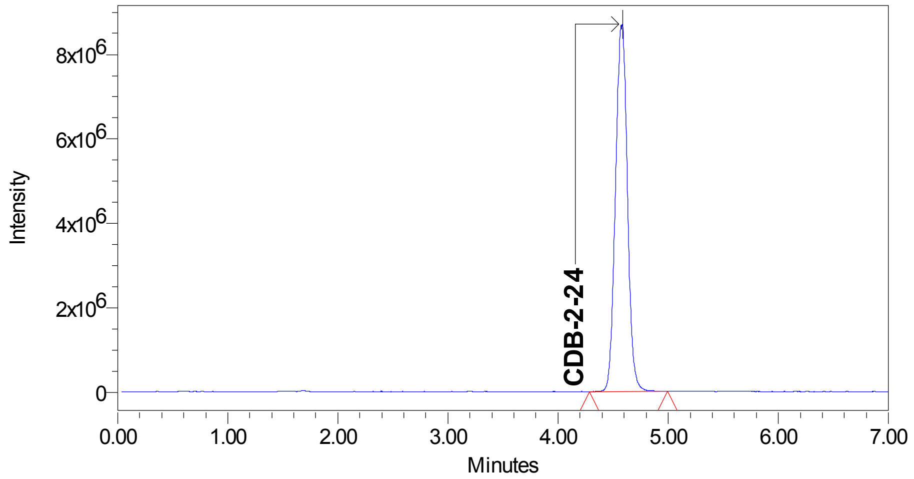

Peak Results

	Name	RT	Area	Height	MS 3D Channel Name
1	CDB-2-24	4.585	65457670	8703762	QDa 1: MS Scan

Match Plot

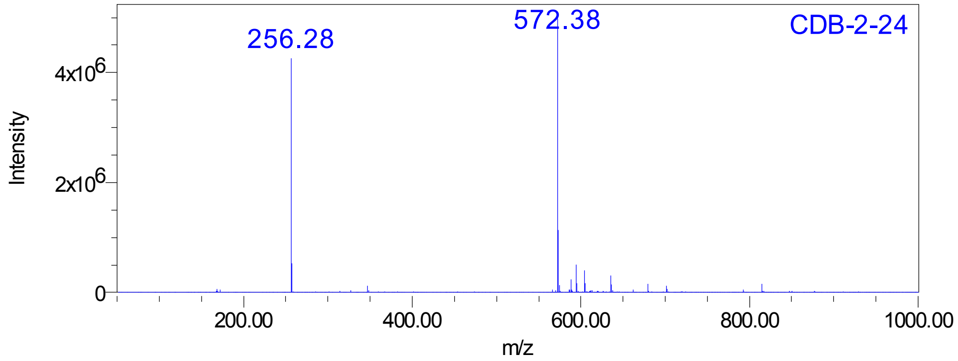

UPLC–ESI–MS data of compound 4b

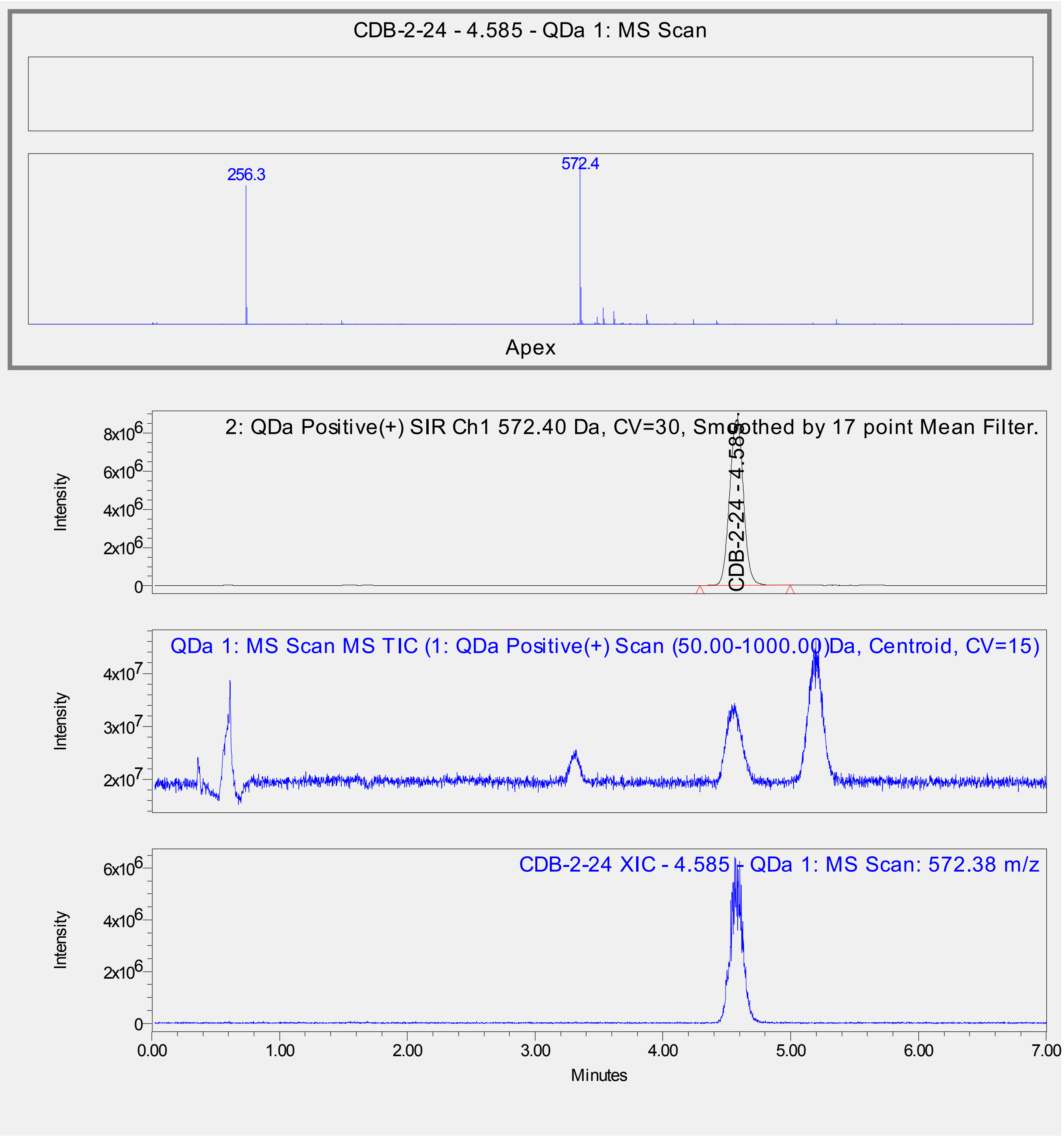

UPLC–ESI–MS data of compound 4b

SAMPLE INFORMATION

Sample Name:	FC-1-113	Acquired By:	
Sample Type:	Unknown	Sample Set Name	001 Prueba 2 FC 1 113 Dil
Vial:	2:C,2	Acq. Method Set:	ID 004 QPDA
Injection #:	1	Injection Volume:	10.00 ul
Run Time:	7.0 Minutes	Date Acquired:	6/4/2021 10:03:55 AM CDT
Column Name:			

ACQUITY UPLC HSS T3 1.8um 3.0 x 100 mm

Auto-Scaled Chromatogram

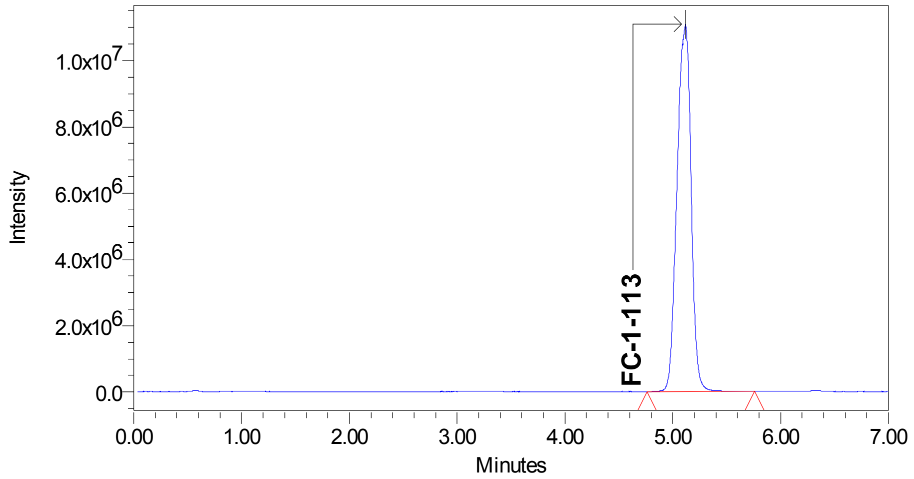

Peak Results

	Name	RT	Area	Height	MS 3D Channel Name
1	FC-1-113	5.118	100670643	11069832	QDa 1: MS Scan

Match Plot

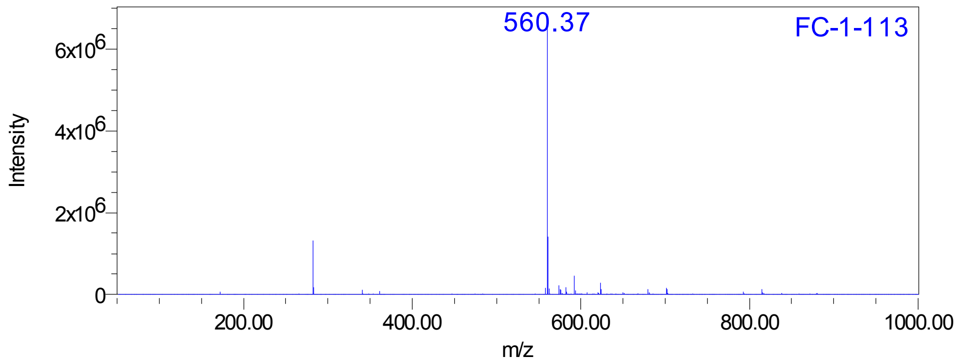

UPLC–ESI–MS data of compound 4c

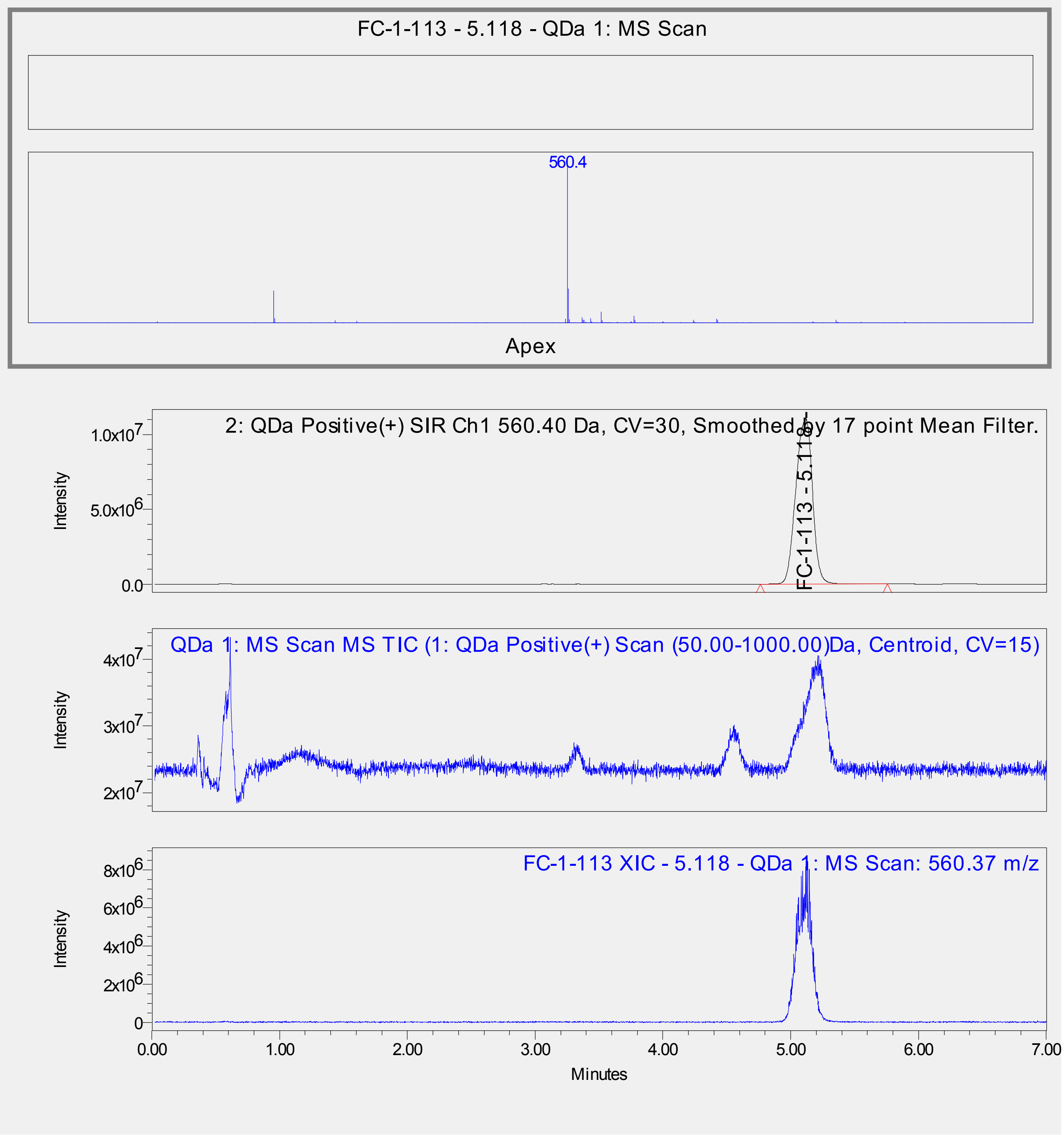

UPLC–ESI–MS data of compound 4c

SAMPLE INFORMATION

Sample Name:	FC-1-121	Acquired By:	
Sample Type:	Unknown	Sample Set Name	001 Prueba 2 FC 1 121
Vial:	2:E,2	Acq. Method Set:	ID 006 QPDA
Injection #:	1	Injection Volume:	10.00 ul
Run Time:	8.0 Minutes	Date Acquired:	6/4/2021 11:03:52 AM CDT
Column Name:			

ACQUITY UPLC HSS T3 1.8um 3.0 x 100 mm

Auto-Scaled Chromatogram

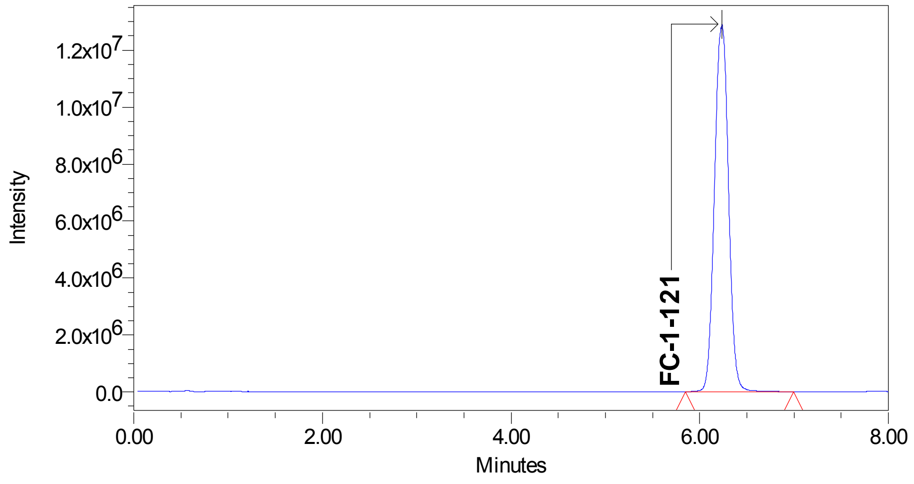

Peak Results

	Name	RT	Area	Height	MS 3D Channel Name
1	FC-1-121	6.235	132861055	12900302	QDa 1: MS Scan

Match Plot

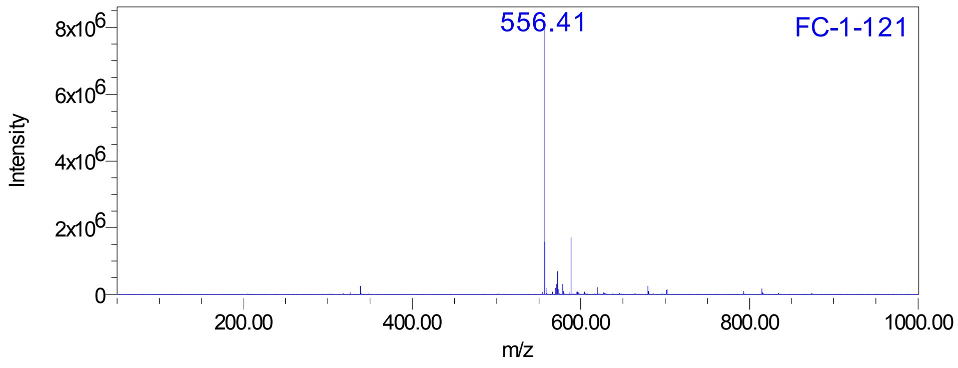

UPLC–ESI–MS data of compound 4d

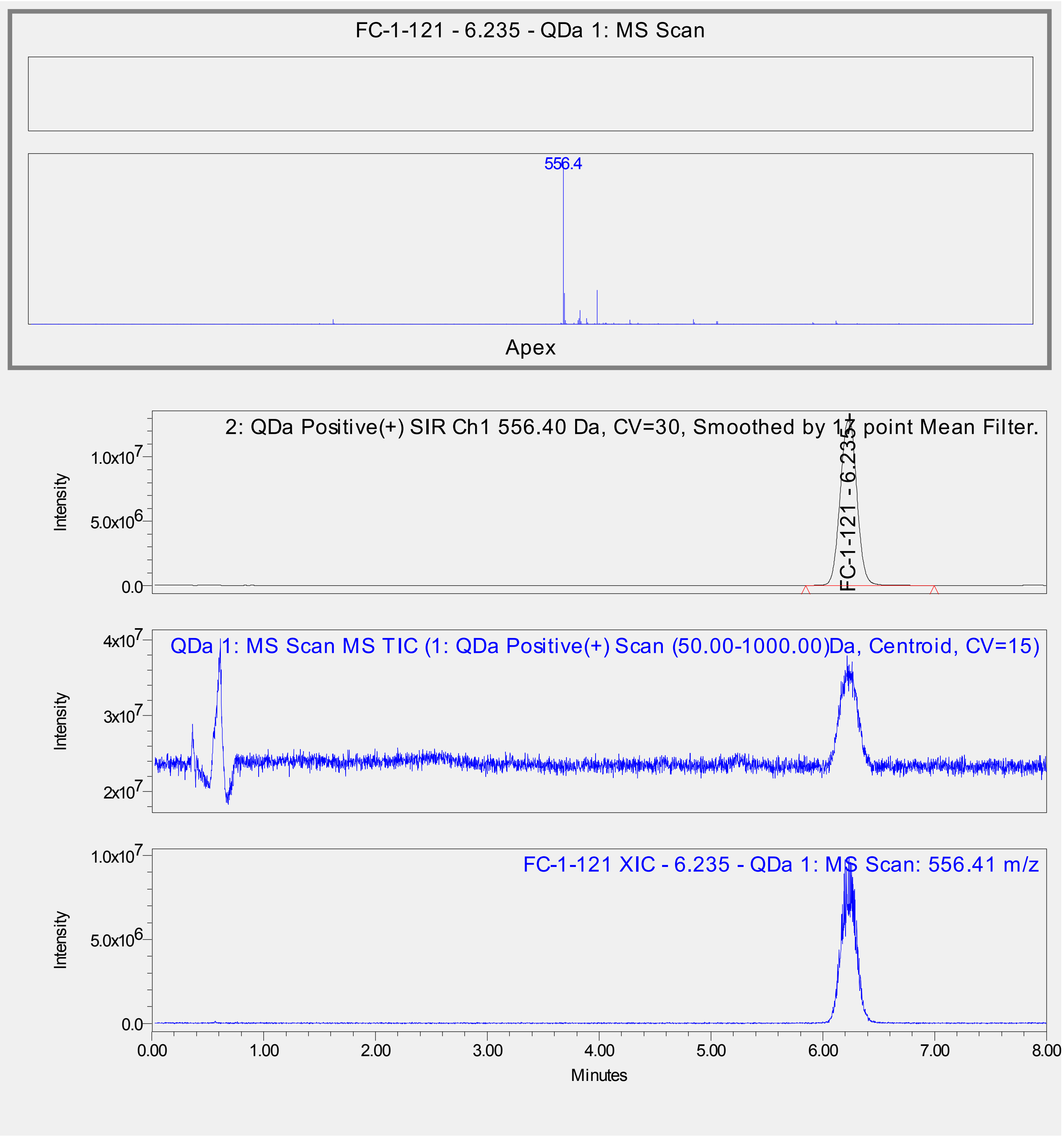

UPLC–ESI–MS data of compound 4d

SAMPLE INFORMATION

Sample Name:	FC-1-109	Acquired By:	
Sample Type:	Unknown	Sample Set Name	001 Prueba 2 FC 1 109 Dil
Vial:	2:D,2	Acq. Method Set:	ID 005 QPDA
Injection #:	1	Injection Volume:	10.00 ul
Run Time:	8.0 Minutes	Date Acquired:	6/4/2021 10:32:00 AM CDT
Column Name:			

ACQUITY UPLC HSS T3 1.8um 3.0 x 100 mm

Auto-Scaled Chromatogram

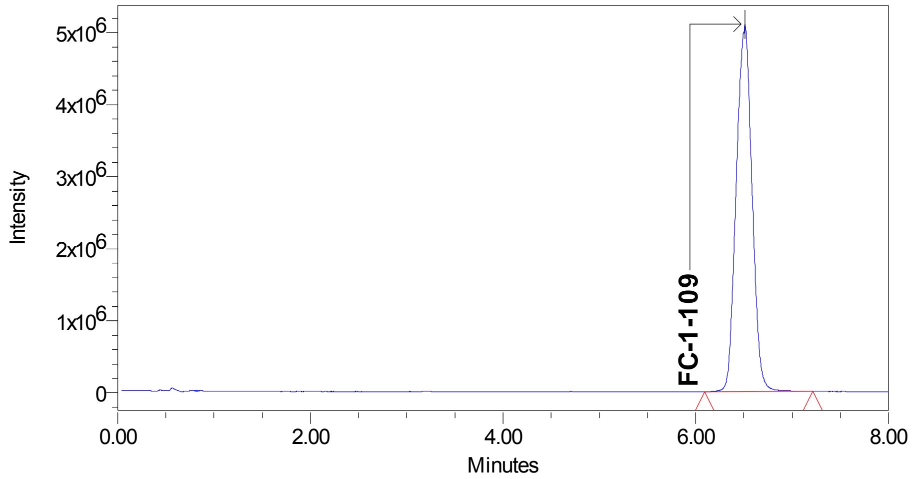

Peak Results

	Name	RT	Area	Height	MS 3D Channel Name
1	FC-1-109	6.510	57968220	5089974	QDa 1: MS Scan

Match Plot

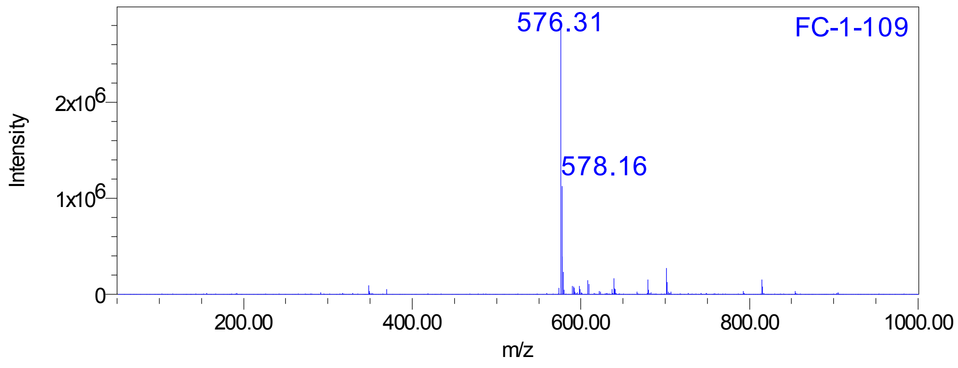

UPLC–ESI–MS data of compound 4e

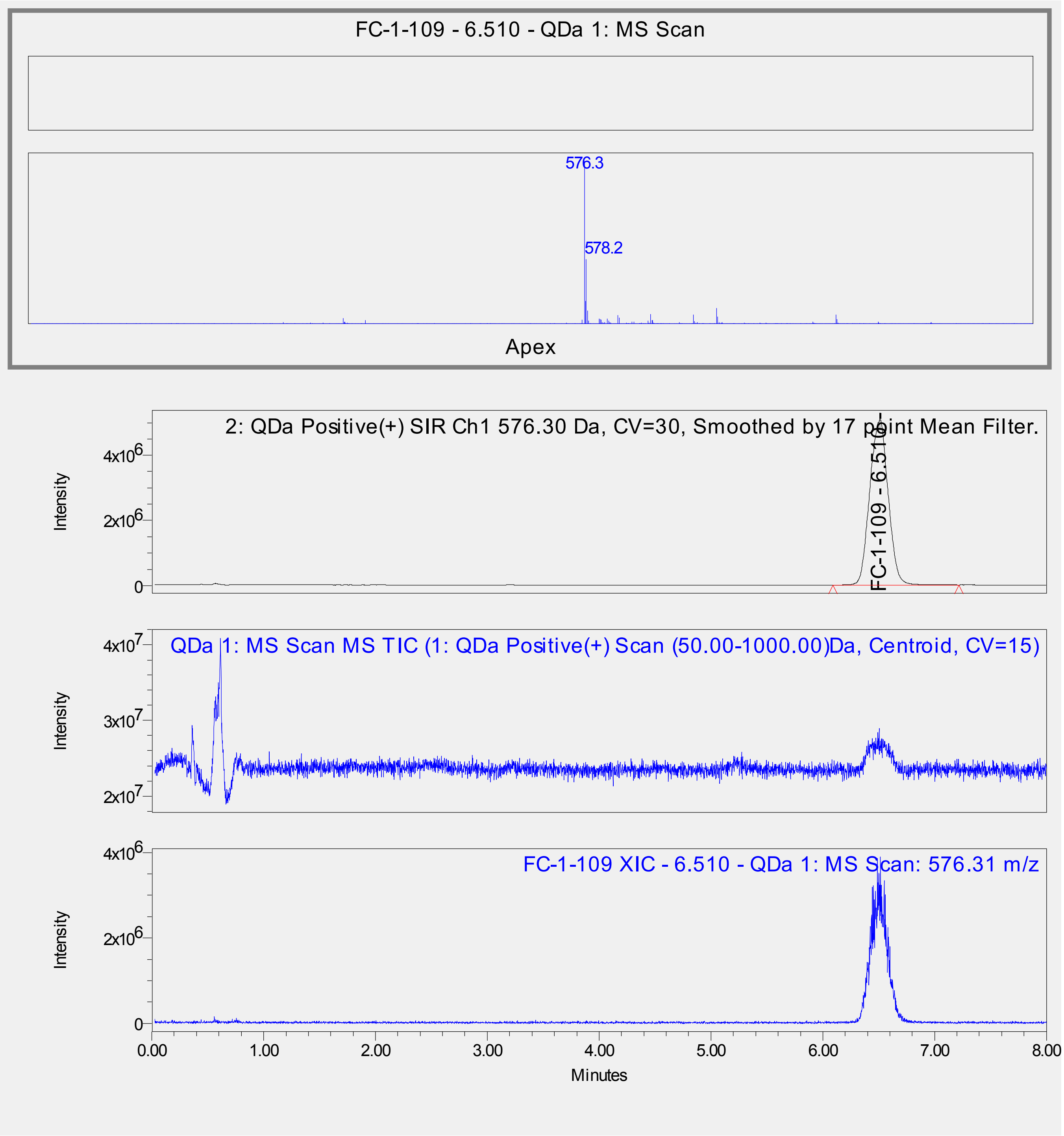

UPLC–ESI–MS data of compound 4e

SAMPLE INFORMATION

Sample Name:	FC-1-114	Acquired By:	
Sample Type:	Unknown	Sample Set Name	001 Prueba 2 FC 1 114 Dil
Vial:	2:F,2	Acq. Method Set:	ID 007 QPDA
Injection #:	1	Injection Volume:	10.00 ul
Run Time:	8.0 Minutes	Date Acquired:	6/4/2021 11:34:13 AM CDT
Column Name:			

ACQUITY UPLC HSS T3 1.8um 3.0 x 100 mm

Auto-Scaled Chromatogram

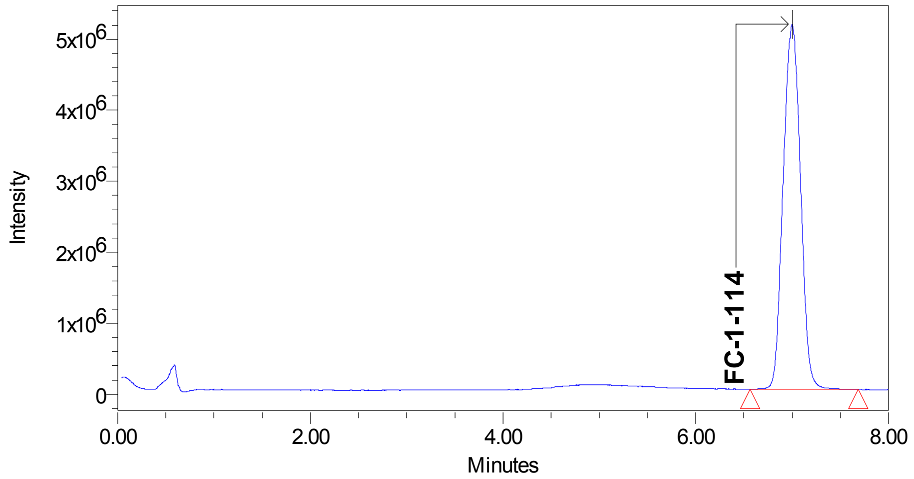

Peak Results

	Name	RT	Area	Height	MS 3D Channel Name
1	FC-1-114	7.001	64379463	5143460	QDa 1: MS Scan

Match Plot

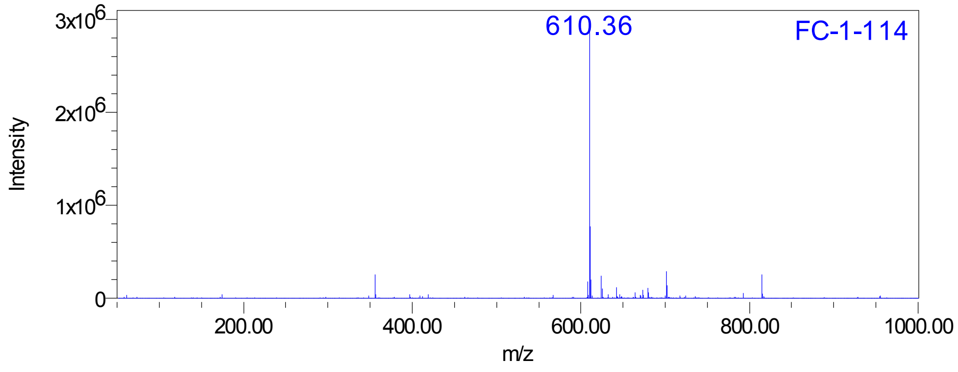

UPLC–ESI–MS data of compound 4f

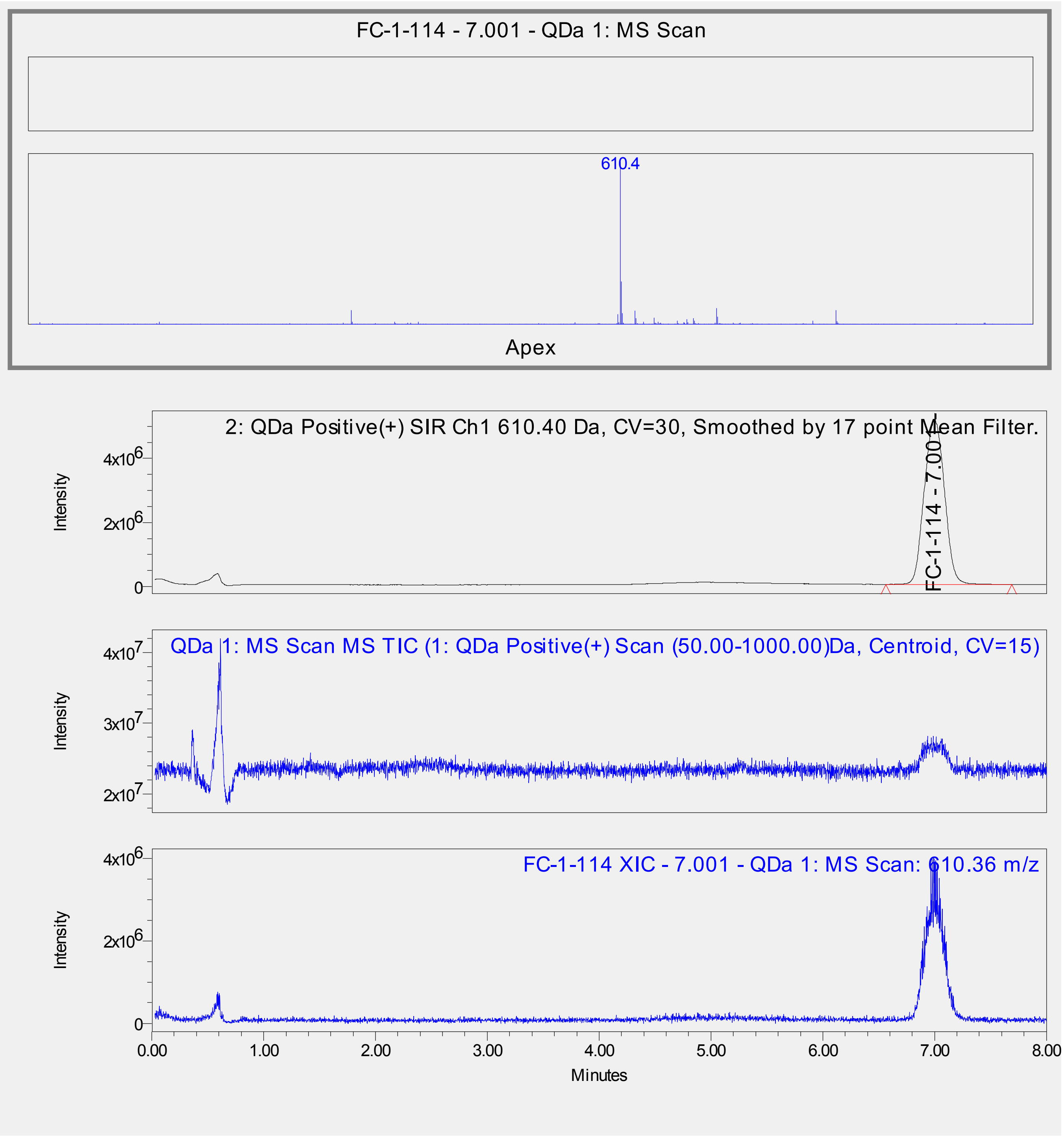

UPLC–ESI–MS data of compound 4f

SAMPLE INFORMATION

Sample Name:	DLC-3-8	Acquired By:	
Sample Type:	Unknown	Sample Set Name	001 Prueba 2 DLC 3 8
Vial:	2:A,3	Acq. Method Set:	ID 008 QPDA
Injection #:	1	Injection Volume:	10.00 ul
Run Time:	8.0 Minutes	Date Acquired:	6/4/2021 12:26:06 PM CDT
Column Name:			

ACQUITY UPLC HSS T3 1.8um 3.0 x 100 mm

Auto-Scaled Chromatogram

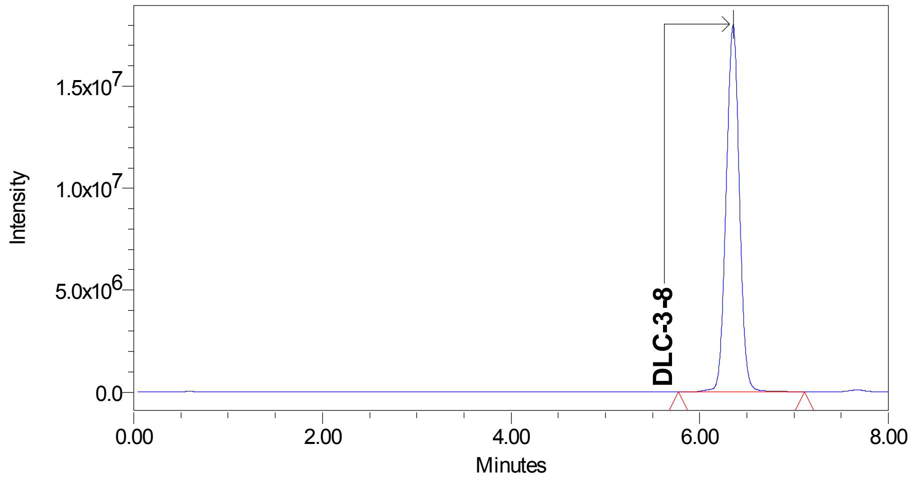

Peak Results

	Name	RT	Area	Height	MS 3D Channel Name
1	DLC-3-8	6.355	175052351	18027727	QDa 1: MS Scan

Match Plot

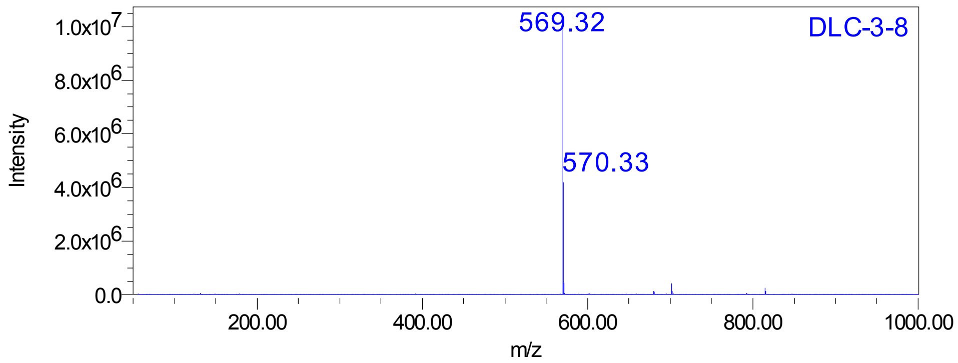

UPLC–ESI–MS data of compound 5a

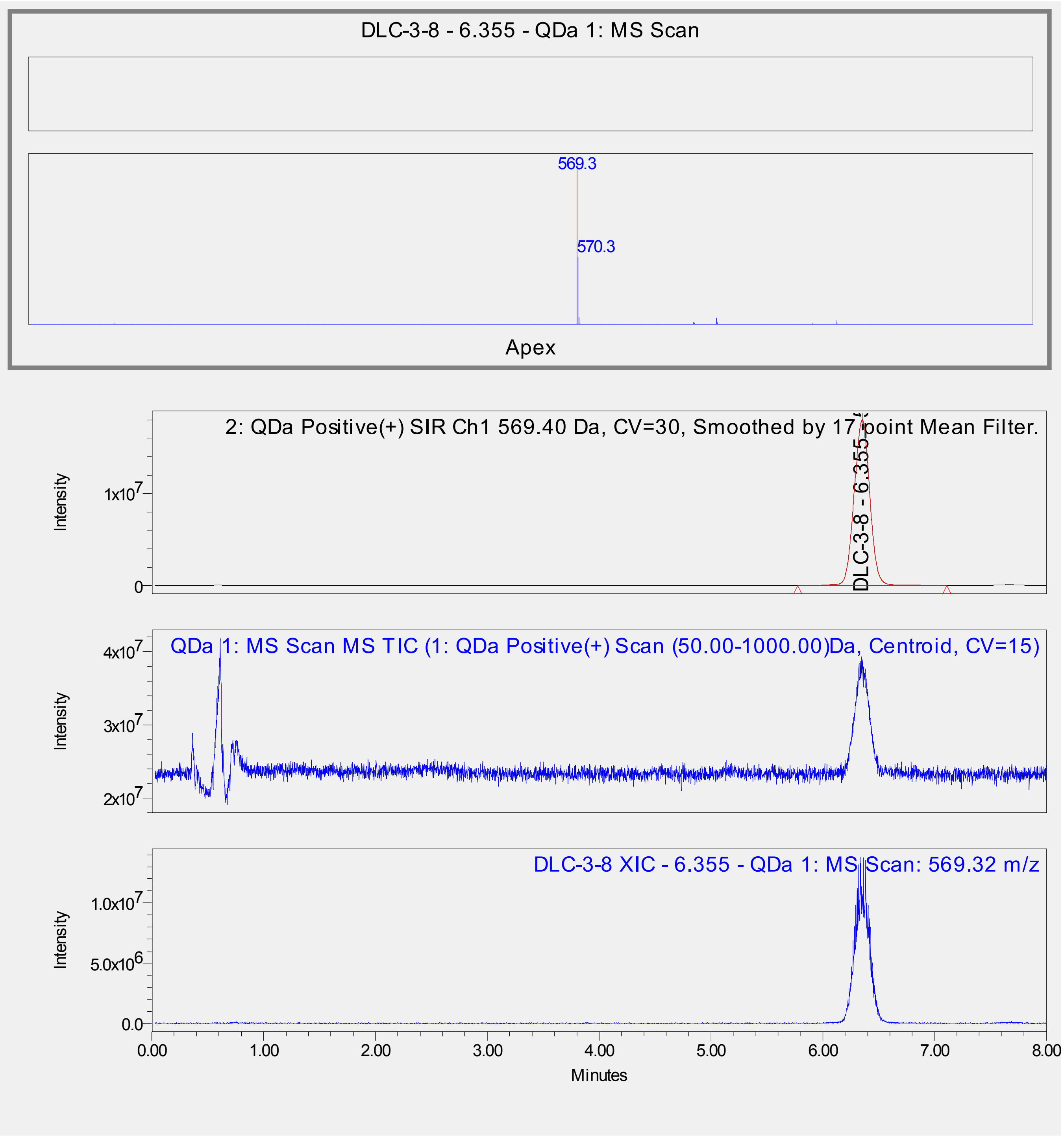

UPLC–ESI–MS data of compound 5b

SAMPLE INFORMATION

Sample Name:	FC-1-122	Acquired By:	
Sample Type:	Unknown	Sample Set Name	001 Prueba 2 FC 1 122
Vial:	2:B,3	Acq. Method Set:	ID 013 QPDA
Injection #:	1	Injection Volume:	10.00 ul
Run Time:	10.0 Minutes	Date Acquired:	6/21/2021 1:19:33 PM CDT
Column Name:			

ACQUITY UPLC HSS T3 1.8um 3.0 x 100 mm

Auto-Scaled Chromatogram

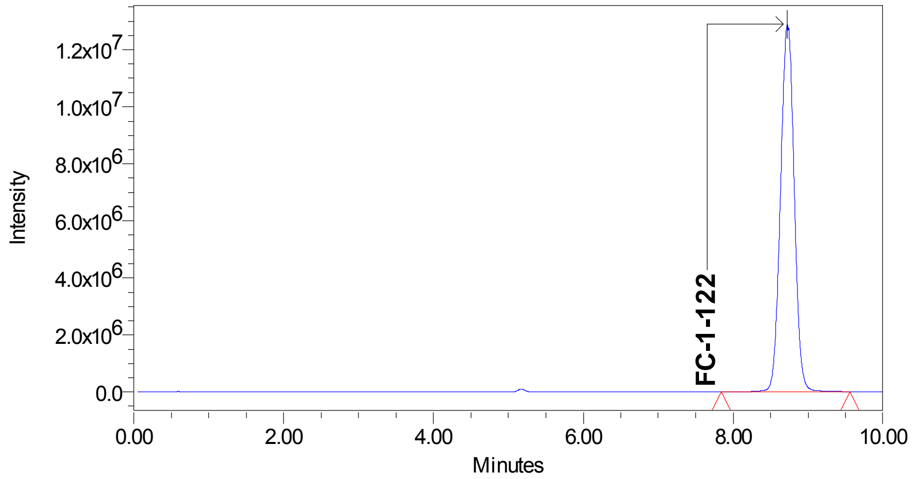

Peak Results

	Name	RT	Area	Height	MS 3D Channel Name
1	FC-1-122	8.721	163186315	12882045	QDa 1: MS Scan

Match Plot

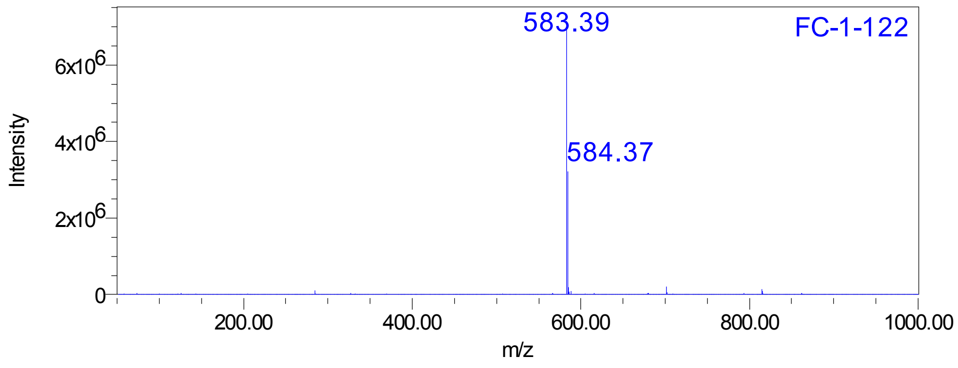

UPLC–ESI–MS data of compound 5c

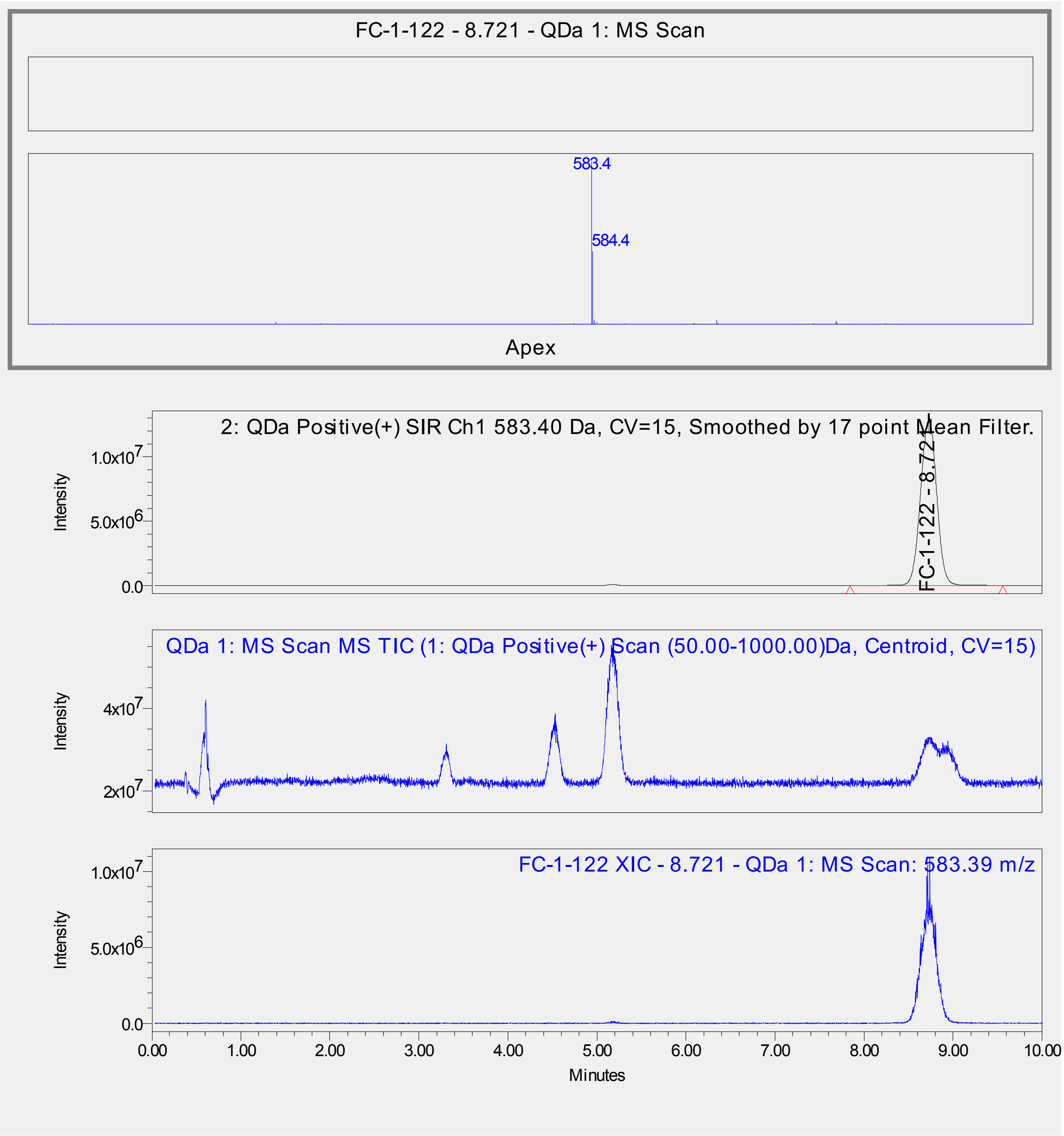

UPLC–ESI–MS data of compound 5c

SAMPLE INFORMATION

Sample Name:	FC-1-115	Acquired By:	
Sample Type:	Unknown	Sample Set Name	001 Prueba 2 FC 1 115
Vial:	2:B,2	Acq. Method Set:	ID 011 QPDA
Injection #:	1	Injection Volume:	10.00 ul
Run Time:	10.0 Minutes	Date Acquired:	6/21/2021 1:08:41 PM CDT
Column Name:			

ACQUITY UPLC HSS T3 1.8um 3.0 x 100 mm

Auto-Scaled Chromatogram

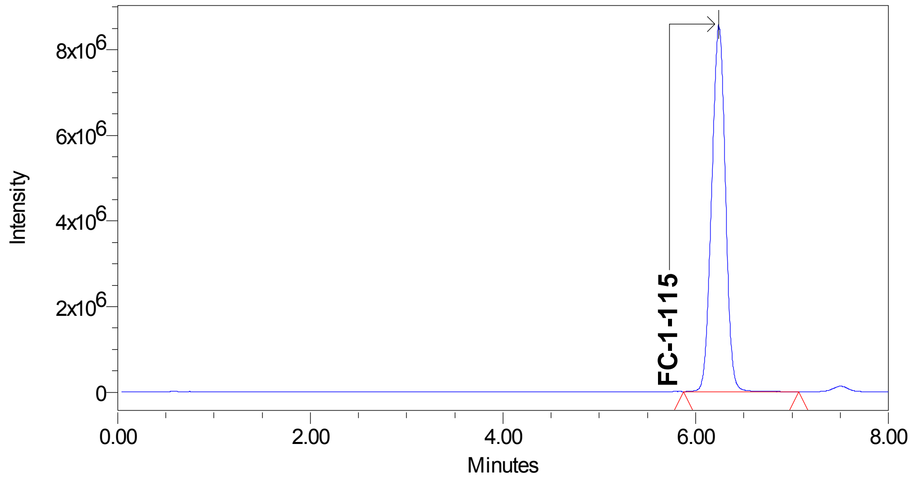

Peak Results

	Name	RT	Area	Height	MS 3D Channel Name
1	FC-1-115	6.238	84701456	8589486	QDa 1: MS Scan

Match Plot

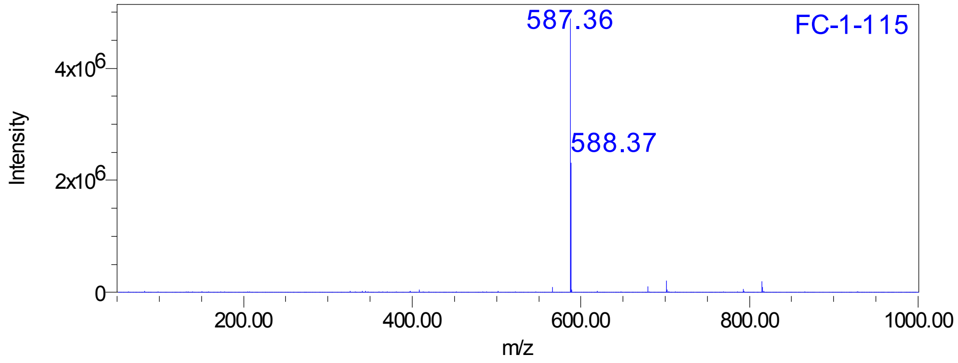

UPLC–ESI–MS data of compound 5d

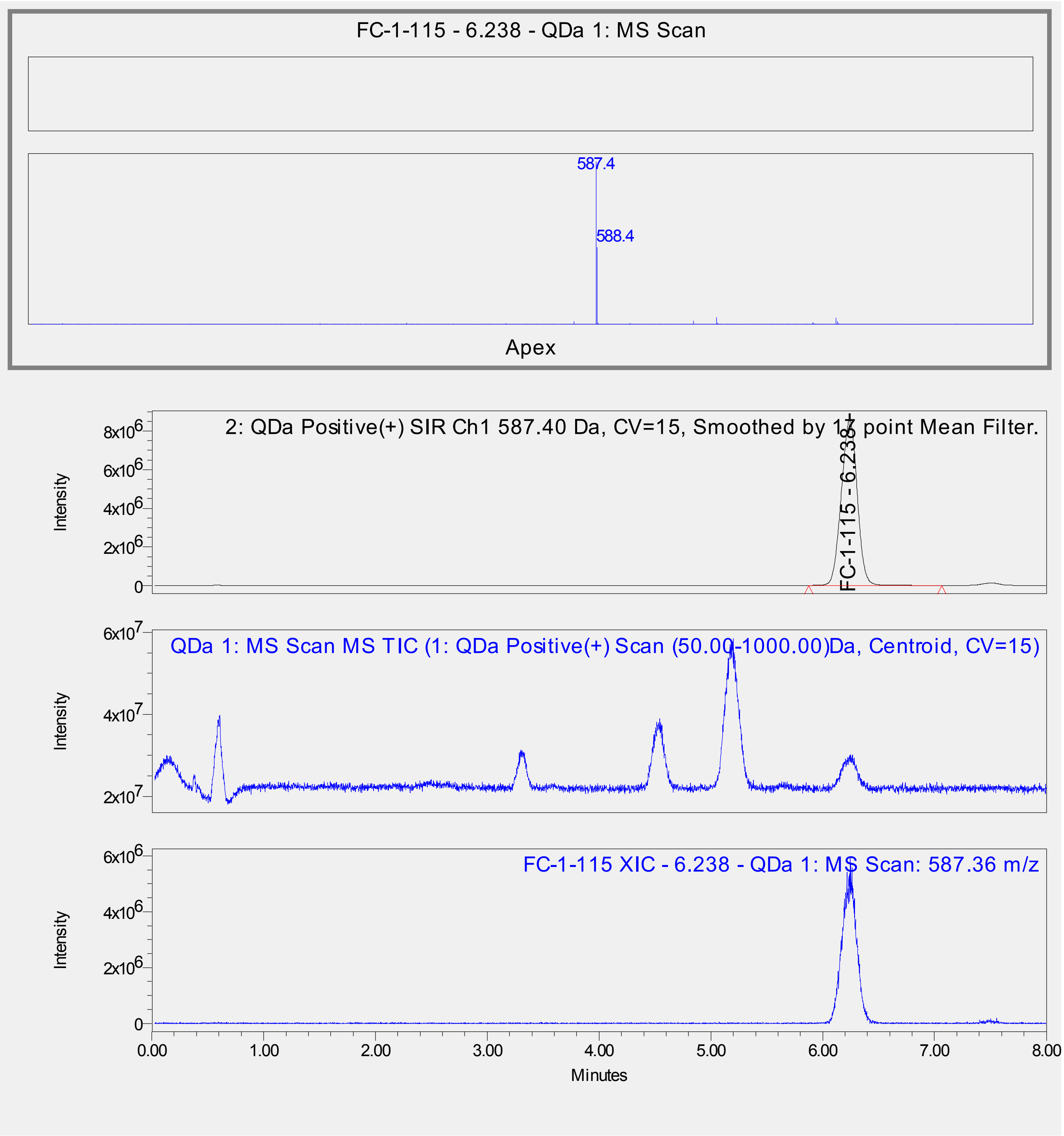

UPLC–ESI–MS data of compound 5d

SAMPLE INFORMATION

Sample Name:	FC-1-119	Acquired By:	
Sample Type:	Unknown	Sample Set Name	001 Prueba 1 FC 1 119
Vial:	2:A,4	Acq. Method Set:	ID 012 QPDA
Injection #:	1	Injection Volume:	10.00 ul
Run Time:	11.0 Minutes	Date Acquired:	6/21/2021 12:37:11 PM CDT
Column Name:			

ACQUITY UPLC HSS T3 1.8um 3.0 x 100 mm

Auto-Scaled Chromatogram

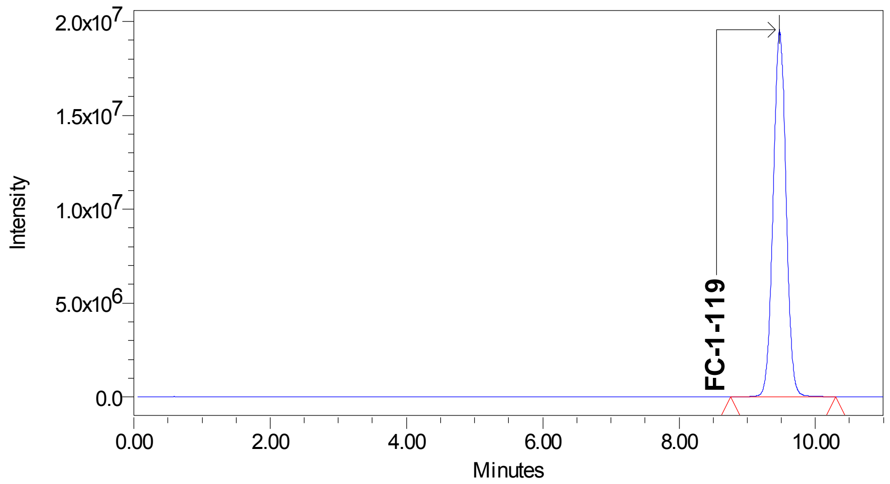

Peak Results

	Name	RT	Area	Height	MS 3D Channel Name
1	FC-1-119	9.472	256951373	19564042	QDa 1: MS Scan

Match Plot

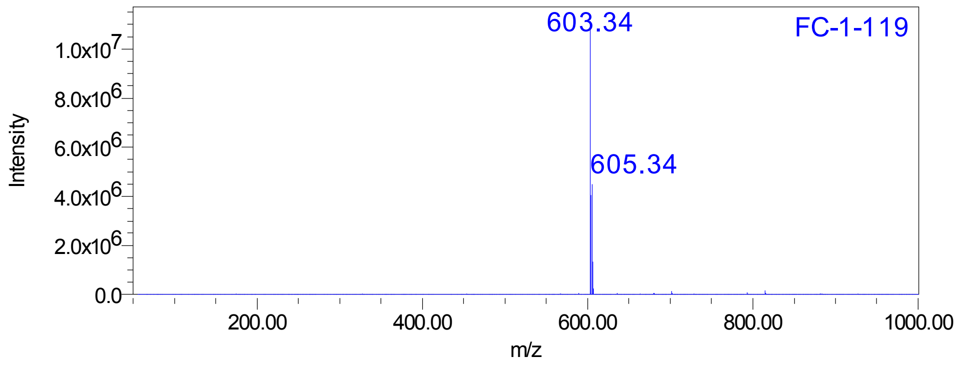

UPLC–ESI–MS data of compound 5e

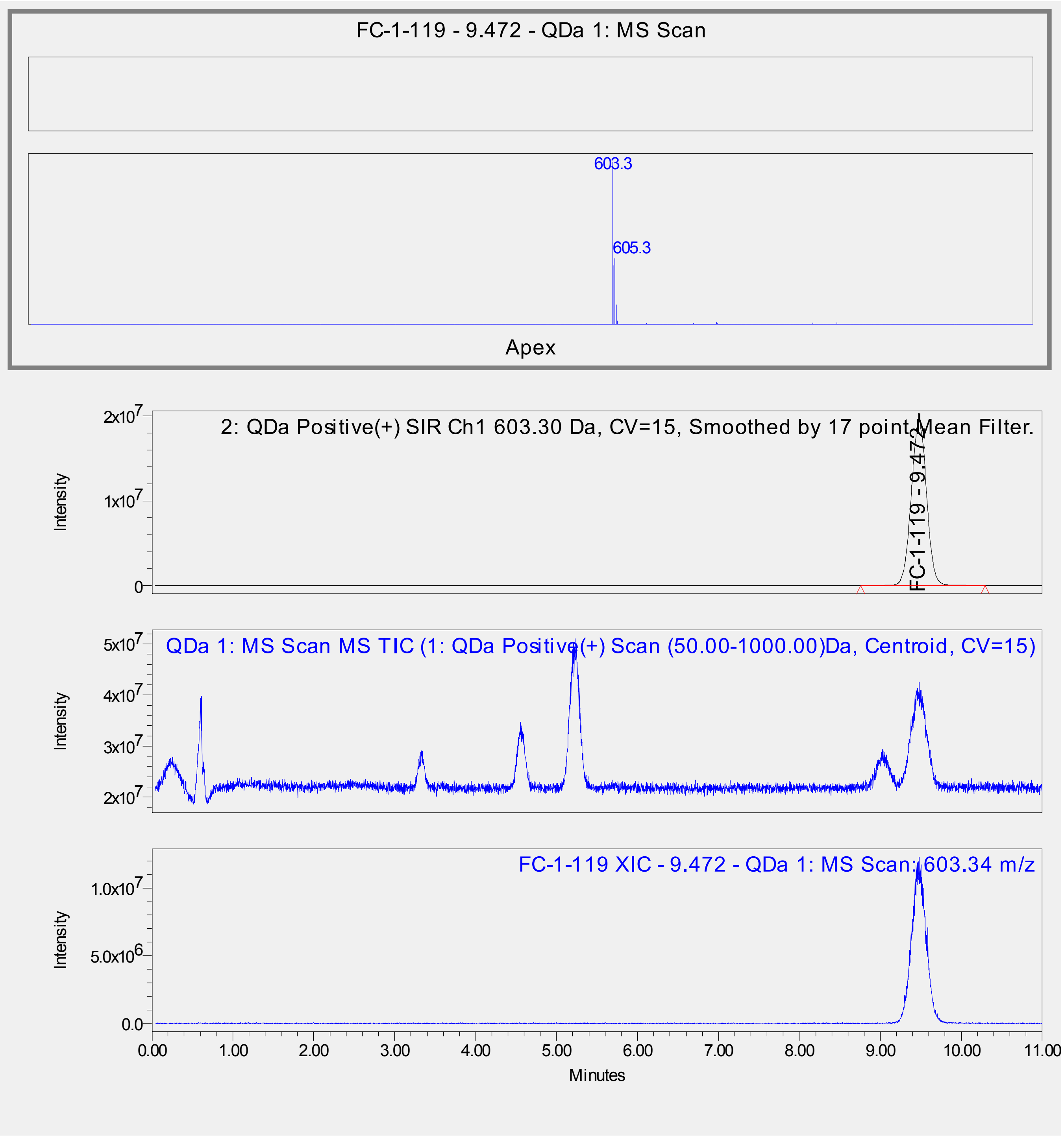

UPLC–ESI–MS data of compound 5e

SAMPLE INFORMATION

Sample Name:	FC-1-116	Acquired By:	
Sample Type:	Unknown	Sample Set Name	001 Prueba 1 FC 1 116
Vial:	2:A,6	Acq. Method Set:	ID 014 QPDA
Injection #:	1	Injection Volume:	10.00 ul
Run Time:	10.0 Minutes	Date Acquired:	6/21/2021 12:49:01 PM CDT
Column Name:			

ACQUITY UPLC HSS T3 1.8um 3.0 x 100 mm

Auto-Scaled Chromatogram

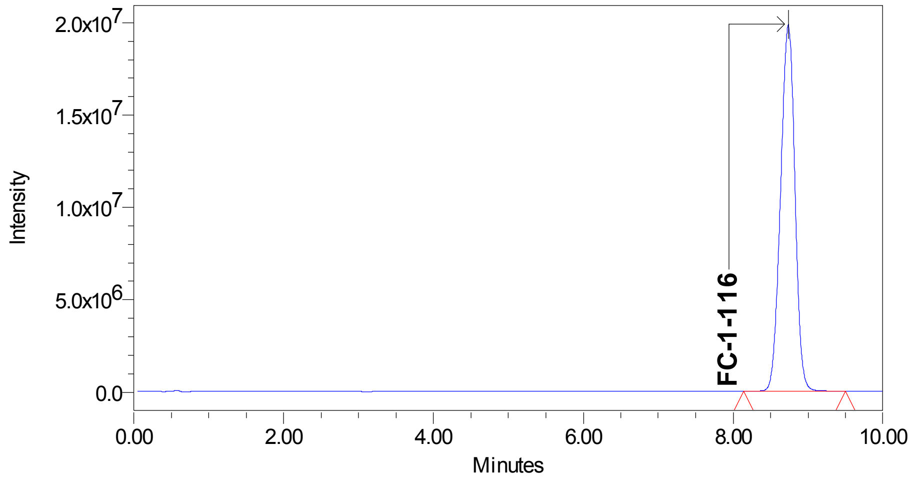

Peak Results

	Name	RT	Area	Height	MS 3D Channel Name
1	FC-1-116	8.737	264967548	19878952	QDa 1: MS Scan

Match Plot

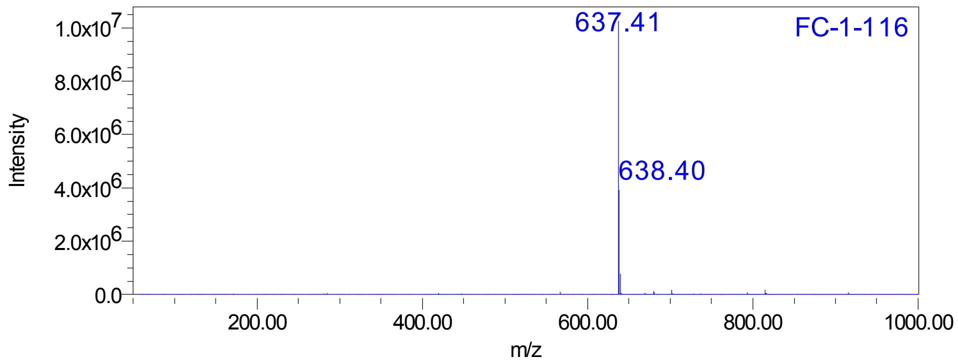

UPLC–ESI–MS data of compound 5f

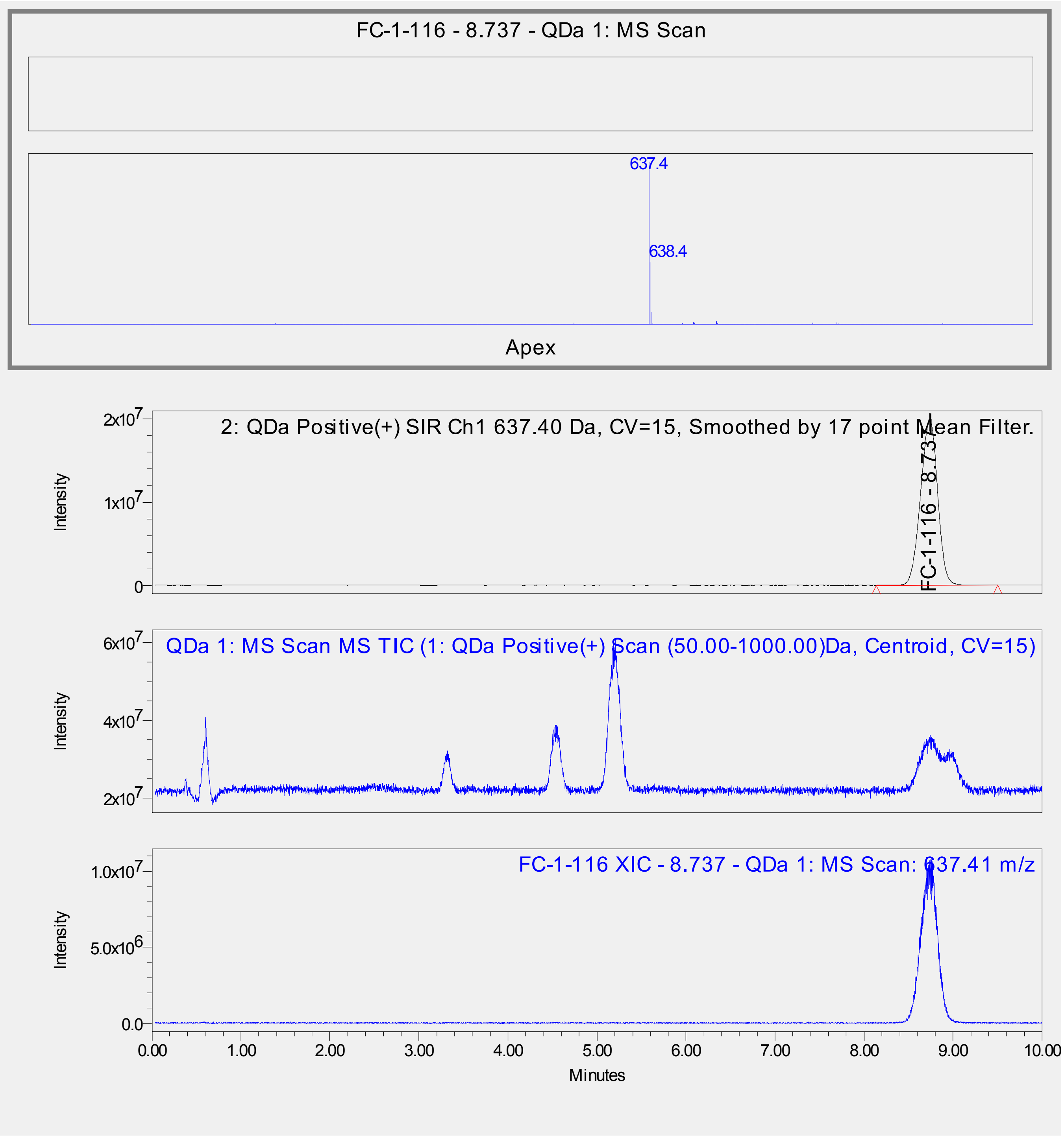

UPLC–ESI–MS data of compound 5f
